# Clinical Symptom Resolution Following PCR-Guided vs. Culture and Susceptibility-Guided Management of Complicated UTI: How Time-To-Antibiotic Start and Antibiotic Appropriateness Mediate the Benefit of Multiplex PCR—An Ad Hoc Analysis of NCT06996301

**DOI:** 10.3390/diagnostics15243107

**Published:** 2025-12-06

**Authors:** Moustafa Kardjadj, Itoe P. Priestly, Roel Chavez, DeAndre Derrick, Thomas K. Huard

**Affiliations:** 1 Dicentra, Toronto, ON M4W 3E2, Canada; 2 Soft Cell Laboratories, Saint George, UT 84770, USA; 3 Doc Lab Inc., Hillsboro, OR 97006, USA; 4 MED-US Consulting, LLC., Austin, TX 78734, USA

**Keywords:** complicated urinary tract infection, multiplex PCR, molecular diagnostics, clinical cure, time-to-antibiotic, antibiotic appropriateness, mediation analysis, randomized trial, diagnostic stewardship, NCT06996301

## Abstract

**Background:** Rapid multiplex PCR assays promise faster and broader detection of uropathogens and resistance markers than conventional quantitative urine culture and susceptibility testing (C&S), but trial evidence linking PCR-guided management to patient-centered outcomes and the mechanisms of any benefit is limited. We performed an ad hoc analysis of the randomized, multicenter NCT06996301 trial to evaluate whether PCR-guided diagnostic management improves clinical symptom resolution in complicated urinary tract infection (cUTI) and to quantify mediation by time-to-antibiotic start and antibiotic appropriateness. **Methods:** Paired PCR and C&S were collected for all participants; treating investigators received and acted on randomized results from one diagnostic modality and remained blinded to the comparator. The modified intention-to-treat (Mod-ITT) cohort at end-of-study (EOS) included 362 participants (PCR *n* = 193; C&S *n* = 169). The primary outcome was complete clinical cure at EOS (absence of all baseline symptoms). Secondary outcomes included partial cure (≥50% symptom reduction) and per-symptom changes. We used mixed-effects logistic regression (site random intercept) to estimate associations, and causal mediation analysis with nonparametric bootstrap (B = 2000) to decompose PCR’s total effect into indirect effects via time-to-antibiotic (log-transformed) and antibiotic appropriateness (binary, adjudicated at EOS) for complete clinical cure and partial cure. **Results:** Median time-to-first antibiotic was substantially shorter in the PCR arm (20 h; IQR 12–36) than in the C&S arm (52 h; IQR 30–66; *p* < 0.001). Antibiotic appropriateness was higher after PCR-guided care (161/193; 83.4%) versus C&S (105/169; 62.1%; *p* < 0.001). Complete clinical cure occurred in 143/193 (74.1%) PCR versus 106/169 (62.7%) C&S (*p* = 0.020); partial cure in 161/193 (83.4%) versus 121/169 (71.6%; *p* = 0.014). In a total-effect mixed model (no mediators), PCR assignment was associated with higher odds of cure (adjusted OR 1.95; 95% CI 1.12–3.39; *p* = 0.018). In the mechanistic model including mediators, antibiotic appropriateness (OR 2.48; 95% CI 1.45–4.24; *p* = 0.001), and time-to-antibiotic (per 1 h, OR 0.95; 95% CI 0.926–0.975; *p* < 0.001) were independently predictive, while the direct arm effect was attenuated (OR 1.10; 95% CI 0.33–3.71). Mediation analysis estimated a statistically significant combined indirect effect (ACME) of 0.0648 (95% CI 0.0343–0.0977), ADE 0.0207 (95% CI −0.0282–0.0784), total effect 0.0796 (95% CI 0.0419–0.1225), and proportion mediated ≈ 74%. Both time-to-antibiotic and appropriateness contributed, with ACME_time ≈ 0.046 and ACME_appropriateness ≈ 0.019. Exploratory analysis using partial cure as the outcome confirmed the robustness and internal validity of the complete-cure findings. **Conclusions**: In this ad hoc analysis of a randomized trial, PCR-guided management of cUTI improved patient-centered symptom outcomes compared with culture-guided care. Most of the benefit was mediated through faster initiation of antibiotics and, to a lesser extent, increased probability of an appropriate initial antibiotic. These results support stewardship-integrated, rapid molecular diagnostics (used alongside culture) to shorten time-to-effective therapy and improve clinical outcomes in cUTI.

## 1. Introduction

Complicated urinary tract infections (cUTIs) are heterogeneous and a frequently severe clinical syndrome in adults that commonly requires empiric antimicrobial therapy while laboratory-based diagnostics are pending. Routine quantitative urine culture and phenotypic antimicrobial susceptibility testing (AST) remain the reference standard for organism identification and MIC (Minimal Inhibitory Concentration) determination, but culture-based workflows are slow and can fail to recover fastidious or low-burden organisms and mixed infections, limitations that reduce the timeliness and actionability of results for time-sensitive clinical decisions. Advances in multiplex molecular diagnostics promise faster, broader detection and early resistance profiling, potentially addressing many of these gaps [1,2,3].

Observational and implementation studies [3,4] show that multiplex PCR panels increase pathogen detection relative to standard culture and susceptibility (C&S), identify polymicrobial infections and some resistance markers, and shorten laboratory turnaround time, features that plausibly enable earlier, more targeted antibiotic prescribing. At the same time, systematic reviews, clinical trials, and guidance documents [5,6,7] emphasize heterogeneous assay performance across platforms and settings and call for careful evaluation of clinical impact, analytic validity, and coverage policy.

The causal chain linking a faster, more sensitive diagnostic to improved patient-centered outcomes is not automatic: rapid identification must be translated into timely and appropriate prescribing to affect symptom resolution. Randomized evidence from other infectious syndromes, especially bloodstream infection, shows that rapid molecular identification combined with antimicrobial stewardship can shorten time to effective therapy and, in some settings, improve clinical outcomes; these studies illustrate both the potential and the operational prerequisites for benefit [8,9,10,11,12,13].

In the UTI context, recent multicenter evaluations of syndromic urinary PCR panels demonstrate substantial gains in detection and changes in prescribing. However, the extent to which these changes produce better symptom outcomes (clinical cure) rather than only process measures is still incompletely characterized. Furthermore, clinical cure (patient-reported symptom resolution) and microbiologic eradication (culture negativity) are related but represent distinct endpoints: improving one does not always guarantee the other, so randomized, patient-centered evaluations that link diagnostics → prescribing → both clinical and microbiologic outcomes are essential to inform practice and policy [14,15,16].

**Figure 1:**
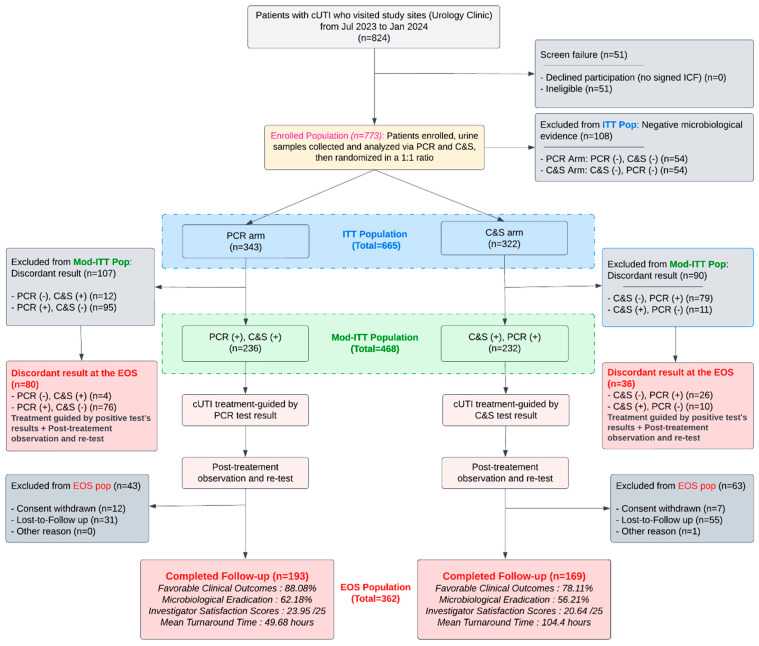
**CONSORT flow diagram of enrollment, randomization, diagnostic testing, study assessments, and primary findings.** All enrolled participants (*n* = 824) first underwent screening for eligibility based on the protocol-defined complicated UTI (cUTI) criteria; 51 individuals did not meet inclusion/exclusion requirements and were classified as screen failures. The remaining 773 participants had paired multiplex PCR and conventional culture and susceptibility (C&S) testing performed at baseline prior to randomization, ensuring a uniform diagnostic starting point for both arms. Participants were then randomized 1:1 into either the PCR-guided arm or the C&S-guided arm. Randomization and allocation concealment were executed automatically through the electronic data capture (EDC) system, which also enforced investigator blinding to the comparator test for the entire study duration. Treating physicians managed patients exclusively using the diagnostic results from their assigned arm and remained blinded to the alternate test results until end-of-study (EOS). Per the parent trial’s protocol design (Supplementary Materials), only concordant-positive cases (PCR+/C&S+) were eligible for inclusion in the primary endpoints of clinical efficacy to preserve randomization integrity and ensure unbiased assessment. This restriction was necessary to preserve the integrity of randomization and blinding: when the assigned diagnostic modality yielded a negative result, investigators were ethically obligated to manage patients based on the other available diagnostic result. In such discordant cases, the assigned arm’s test result could not guide treatment, breaking operational blinding and preventing a valid randomized comparison. The discordant cases (PCR+/C− and PCR−/C+) were analyzed separately, as secondary endpoints. All participants underwent repeat PCR and C&S testing at EOS. Investigator satisfaction (21 physicians, 6 sites) was assessed at EOS using a structured questionnaire evaluating clarity of interpretation, timeliness, perceived clinical impact, and antibiotic appropriateness relative to the comparator modality. Turnaround time (TAT) was recorded from urine collection to availability of diagnostic results, allowing a direct comparison of molecular versus culture workflows. Favorable clinical outcome (the primary endpoint of the parent trial) was defined as resolution of at least one baseline symptom without emergence of new symptoms. Microbiological eradication (the secondary endpoint) was defined as absence at EOS of all baseline uropathogens on quantitative culture.

The randomized, multicenter NCT06996301 trial [7,8,9] was designed to address exactly this gap by performing paired PCR and culture testing on all participants (baseline and end of study) and randomizing treating investigators to act on results from one diagnostic modality while remaining blinded to the comparator until end-of-study (EOS). In its primary analysis, the trial demonstrated that PCR-guided testing significantly reduced diagnostic turnaround time (mean 49.68 h vs. 104.4 h with C&S; *p* < 0.001), enabling earlier treatment modifications and yielding higher clinician satisfaction scores (23.95 ± 1.96 vs. 20.64 ± 4.12; *p* < 0.001). Furthermore, favorable clinical outcome rates (the resolution of at least one baseline symptom) were higher with PCR (88.08% vs. 78.11%; *p* = 0.011), with the greatest benefit observed among higher-risk patients, including older adults, females, and those with polymicrobial infections. The primary findings of NCT06996301 were reported previously [7,8,9] and summarized in Figure 1.

Building directly on the primary analysis of NCT06996301, which established faster diagnostic turnaround, earlier treatment modification, and improved clinician-assessed outcomes in the PCR arm, we designed the present ad hoc analysis to investigate how these diagnostic advantages translate into patient-centered clinical cure. In this ad hoc analysis of NCT06996301, we therefore pursue the following three linked objectives: (1) compare complete clinical cure and partial cure between PCR-guided and culture-guided management; (2) identify independent predictors of complete clinical cure and partial cure including diagnostic arm, time-to-antibiotic, microbiologic eradication, and antibiotic appropriateness; and (3) quantify, using causal mediation methods, the extent to which any PCR-associated improvement in complete clinical cure and partial cure is mediated through earlier antibiotic initiation and through increased likelihood of an appropriate initial antibiotic. The trial collected the timing of antibiotic initiation prospectively, structured investigator adjudication of antibiotic appropriateness, and patient-level symptom data—features that enable not only arm-level outcome comparisons but also formal causal mediation analyses to quantify the pathways through which diagnostic assignment influences clinical cure (for example, via earlier antibiotic start and/or improved antibiotic appropriateness). We hypothesized that PCR-guided management would increase complete clinical cure and partial cure relative to C&S and that the bulk of that benefit would be explained by accelerated initiation of effective therapy with a secondary contribution from improved initial antibiotic appropriateness driven by broader pathogen and resistance detection. Demonstrating and quantifying these pathways has direct implications for diagnostic stewardship, operational design, and policy decisions about adopting rapid molecular diagnostics for cUTI.

## 2. Methods

### 2.1. Study Design and Ethics

This is an ad hoc secondary analysis of the randomized, parallel, investigator-blinded, multicenter clinical trial NCT06996301 conducted in 2023–2024 [7,8,9]. The parent trial compared a rapid multiplex PCR panel (DOC Lab UTM 2.0) with conventional quantitative urine culture and susceptibility testing (C&S) for management of complicated urinary tract infection (cUTI) in adults across six geographically distinct U.S. clinical sites (Augusta, Albany, Norman, Phoenix, Silicon Valley, Southeastern) in an outpatient setting. The trial was registered at ClinicalTrials.gov (https://clinicaltrials.gov/study/NCT06996301?term=NCT06996301&rank=1, accessed on 5 December 2025). and conducted under the ethical oversight and IRB approval reported in the parent protocol (Advarra IRB, Pro00071764; approval date 22 May 2023). dicentra CRO performed trial conduct, monitoring, and data management. A CONSORT flow diagram describing screening, randomization, paired testing, and the analytic populations is provided in Figure 1. For transparency and reproducibility, the full study protocol has been included as Supplementary Materials, along with detailed descriptions of laboratory procedures and analytic workflows used throughout the trial.

### 2.2. Analytic Cohorts and Sample Selection

Two analytic sets from the trial database were used, depending on the analysis objective:

1. *Descriptive and per-symptom analyses (symptoms resolution):* the end-of-study (EOS) population, including all participants with completed clinical follow-up (28 days) and paired PCR+ culture results (*N* = 362; PCR arm *n* = 193, C&S arm *n* = 169). Discordant EOS specimens (PCR+/C− and PCR−/C+) were analyzed as a nested subgroup (*N* = 116; PCR+/C− = 102; PCR−/C+ = 14).
2. *Multivariable prediction and causal mediation analyses*: the end-of-study (EOS) population, including all participants with completed clinical follow-up (28 days) and paired PCR + culture results (*N* = 362; PCR arm *n* = 193, C&S arm *n* = 169).

Inclusion and exclusion criteria followed the parent trial [8] and were detailed in the study protocol (Supplementary Materials); notably, no participants had documented antibiotic exposure in the 48 h prior to enrollment per trial protocol.

### 2.3. Data Sources and Variables

An ad hoc extract of the trial database (CSV plus data dictionary) was used. The following variables were available and used in analyses:

- Outcome variables
  ∘ *Complete clinical cure (primary outcome, binary)*: absence of all baseline symptoms recorded at the prespecified EOS visit.
  *Partial cure (secondary, binary)*: ≥50% reduction in number of baseline symptoms at EOS.
  *Per-symptom presence/absence* at enrollment and EOS (dysuria/frequency/urgency/hematuria, suprapubic/pelvic pain, costovertebral angle [CVA] tenderness, fever, nausea/vomiting, leukocyturia/pyuria).
  *Microbiologic eradication* at EOS: absence of baseline pathogen (*culture−*) in the C&S Arm and (PCR−) in the PCR Arm.

- Exposure/predictors/covariates
  ∘ Randomized diagnostic arm (PCR vs. C&S).
  *Time-to-antibiotic start* (time_to_abx_h; hours): defined as the interval from urine collection to administration of the first antibiotic prescribed based on the assigned diagnostic results. **Note:** *Empiric antibiotics administered at the baseline visit in the culture arm (per standard-of-care practice) were **not** included in this calculation. time_to_abx_h was* used as both predictor and mediator. For mediation, we used the log-transform to stabilize skew: ${\log\text{\_}\mathrm{time}}_{i}=log({\mathrm{time}\text{\_}\mathrm{to}\text{\_}\mathrm{abx}\text{\_}h}_{i}+1)$
  *Antibiotic* appropriateness (binary variable: Yes/No): Antibiotic appropriateness (binary variable: Yes/No): assessed by the treating investigator at end-of-study (EOS), once diagnostic blinding was lifted, and used as both predictor and mediator. Appropriateness was defined by comparing the antibiotic prescribed (based on the assigned diagnostic) with (a) the complete pathogen and resistance profile detected by both diagnostics and (b) antimicrobial treatment guidelines.
    - Appropriate (Yes): the prescribed antibiotic, based on assigned diagnostic arm results, provided adequate coverage for all uropathogens and resistance markers and did not omit any organism or marker detected by the comparator test.
    Inappropriate (No): the prescribed antibiotic failed to cover one or more organisms or resistance markers detected by the comparator test.
  Baseline symptom count (2, 3, or 4).
  Mono- vs. polymicrobial infection (culture/PCR concordance flag).
  Age category (≥65 vs. <65 years), sex at birth (male/female), presence of comorbidity (metabolic disease, immunosuppression, CKD), recurrent UTI status (≥3 UTI episodes in prior 12 months), and failure of first-line therapy before enrollment.

All variable definitions and coding rules were reported in a study data dictionary. Time stamps (collection time, lab receipt, antibiotic administration) were used to calculate time intervals and ensure temporal ordering for mediation.

### 2.4. Derived Variables and Outcome Classification

- *Antibiotic appropriateness:*

Antibiotic appropriateness was derived from the treating investigator’s end-of-study (EOS) questionnaire, specifically question #5: “*Compared to the comparator test, the clinical decision (antibiotic choice) made with the assigned test result is appropriate and has yielded better patient clinical outcomes.*” Responses were reclassified as follows:

- Appropriate: if the investigator selected Agree or Strongly agree.
- Inappropriate: if the investigator selected *Neutral*, *Disagree*, or *Strongly disagree.*

This variable was used both descriptively and analytically as a binary indicator (Yes/No) in regression and mediation models to assess its influence on clinical cure and to quantify its mediating role between diagnostic arm and outcome.

- *Percent symptoms change* for each symptom category ():

$$\%\;\mathrm{change}=(\frac{\mathrm{EOS}\;\mathrm{count}}{\mathrm{Baseline}\;\mathrm{count}}-1)\times 100$$

Negative values denote symptom reduction.

- *Symptom-change categories*: subject-level change collapsed into clinically interpretable bins:
  ∘ *Clinical failure*: no symptom reduction or addition of symptoms (0 symptom reduction or ≥1 additional symptom).
  *Favorable clinical outcome (FCO)*: ≥1 symptom reduction, further subdivided into 1, 2, 3, or 4 symptom reductions.
- *Discordant specimen definitions*:
  ∘ *PCR+/C−*: PCR detects a pathogen at baseline, whereas culture is negative for that pathogen.
  *PCR−/C+*: culture detects organism at baseline not detected by PCR.

### 2.5. Statistical Analysis

Analyses were performed in R (version 4.5.2). We report two-sided *p*-values and 95% confidence intervals (CIs). Statistical significance was assessed at α = 0.05 unless otherwise stated.

- *Descriptive statistics and per-symptom testing*

Counts and percentages were reported for categorical variables; medians and interquartile ranges (IQR) for continuous variables. Between-arm comparisons used Pearson’s χ^2^ tests for categorical variables when all expected cell counts ≥5; Fisher’s exact test was used otherwise (two-sided). For baseline, EOS comparisons, we compared prevalence by arm using χ^2^/Fisher tests; percent changes were computed as above and displayed for interpretability.

- *Multivariable prediction model (logistic mixed effects)*

To identify independent predictors of complete clinical cure (absence of all baseline symptoms at EOS), we fitted pre-specified multivariable logistic mixed-effects models with a random intercept for clinical site to account for clustering and between-site practice variation.

General model form:

$$\mathrm{logit}(P({\mathrm{cure}}_{i}))=\beta_{0}+\beta_{1}\;{\mathrm{Arm}}_{i}+\beta_{2}\;{\log\text{\_}\mathrm{time}}_{i}+\beta_{3}\;{\mathrm{Microerad}}_{i}+\beta_{4}\;{\mathrm{Appropriate}}_{i}+\sum_{k=5}^{K}\beta_{k}X_{ik}+u_{\mathrm{site}(i)}$$

where

∘ ${\mathrm{Arm}}_{i}$ = 1 if randomized to PCR-guided management, 0 if C&S-guided;
${\log\text{\_}\mathrm{time}}_{i}=log({\mathrm{time}\text{\_}\mathrm{to}\text{\_}\mathrm{abx}\text{\_}h}_{i}+1)$, natural-log transform of hours from specimen collection to first antibiotic (stabilizes skew);
${\mathrm{Microerad}}_{i}$ = 1 if culture negative at EOS (microbiologic eradication), 0 otherwise;
${\mathrm{Appropriate}}_{i}$ = 1 if first prescribed antibiotic was adjudicated appropriate (Yes) at EOS, 0 if inappropriate;
$X_{ik}$ = baseline covariates: age category (≥65 vs. <65), sex at birth, presence of comorbidity (metabolic disease/immunosuppression/CKD), recurrent UTI (≥3 prior episodes), failure of first-line therapy before enrollment, baseline symptom count (2, 3, or 4), infection type (mono vs. poly);
$u_{\mathrm{site}(i)}∼N\left(0,\sigma_{\mathrm{site}}^{2}\right)\;$ is a site random intercept.

Modeling strategy and objectives

∘ *Model T (Total-effect):* estimate the total effect of randomized assignment (Arm) on clinical cure. This model *did not include* post-randomization mediators (log_time, Appropriate) when presenting the total effect. Model T, therefore, includes Arm and the baseline covariates $X_{ik}$ plus site random intercept.
*Model M1 (time mediation check):* included log_time (mediator) with baseline covariates, quantified attenuation of the Arm coefficient when adjusting for time.
*Model M2 (mechanistic model):* includes both mediators log_time and Appropriate plus baseline covariates, used to show associations conditional on mediators and to construct mediation decomposition.

Models were fit with glmer() (lme4) using the “bobyqa” optimizer and increased iteration limits when necessary. Fixed-effect coefficients were exponentiated to adjusted odds ratios (ORs) with 95% Wald CIs. For robustness we computed cluster-robust sandwich standard errors with the clubSandwich package and inspected variance inflation factors (VIF) for multicollinearity (flagging VIF > 5). We preserved all prespecified covariates (no data-driven variable selection). We verified the rule-of-thumb of ≥10 outcome events per parameter to reduce overfitting risk.

- *Causal mediation analysis (decomposition of indirect effects)*

We used causal mediation analysis to decompose the total effect of Arm into indirect pathways mediated by (a) time-to-antibiotic (log_time) and (b) antibiotic appropriateness (Appropriate). Two complementary mediation strategies were implemented [17,18]:

1. Parallel mediation (time and appropriateness as independent mediators): estimate the separate indirect effects of Arm → time → cure and Arm → appropriateness → cure, and the combined indirect effect.
2. Sequential mediation (time upstream of appropriateness): appropriate when earlier availability of results plausibly increases the probability of prescribing an appropriate antibiotic (Arm → time → appropriateness → cure). In this specification we model appropriateness as potentially dependent on log_time.

Models used for mediation

*Mediator model 1 (continuous mediator: log_time)*

$${\log\text{\_}\mathrm{time}}_{i}=\alpha_{0}+\alpha_{1}\;{\mathrm{Arm}}_{i}+\sum_{k}\alpha_{k+1}X_{ik}+b_{\mathrm{site}(i)}+\varepsilon_{i},b_{\mathrm{site}}∼N(0,\tau^{2}),\;\varepsilon ∼N(0,\sigma^{2}).$$

*Mediator model 2 (binary mediator: Appropriate)*.

For binary appropriateness, we used a logistic mixed model:

$$\mathrm{logit}(P({\mathrm{Appropriate}}_{i}=1))=\delta_{0}+\delta_{1}\;{\mathrm{Arm}}_{i}+\delta_{2}\;{\log\text{\_}\mathrm{time}}_{i}+\sum_{k}\delta_{k+2}X_{ik}+b_{\mathrm{site}(i)}^{\prime }.$$

*Outcome model (binary cure; logistic mixed model)*:

$$\mathrm{logit}(P({\mathrm{cure}}_{i}))=\gamma_{0}+\gamma_{1}\;{\mathrm{Arm}}_{i}+\gamma_{2}\;{\log\text{\_}\mathrm{time}}_{i}+\gamma_{3}\;{\mathrm{Appropriate}}_{i}+\sum_{k}\gamma_{k+3}X_{ik}+u_{\mathrm{site}(i)}.$$

All mediator and outcome models include identical baseline covariates $X_{ik}$ and site random intercepts.

*Estimation and inference:*

We estimated ACME (average causal mediation effect; indirect effect), ADE (average direct effect), total effect, and proportion mediated using the mediate() function in the mediation R package with nonparametric bootstrap (B = 2000) to produce percentile 95% CIs. For sequential mediation (Arm → log_time → Appropriate → cure) we implemented chained mediation by first modeling log_time, then appropriateness conditional on log_time, and finally the outcome conditional on both mediators; furthermore, we used a combination of the mediate() calls and structural equation modeling as sensitivity. We report point estimates and bootstrap 95% CIs. Sensitivity to unmeasured mediator–outcome confounding was evaluated using medsens() [17,18].

*Interpretation rules:*

∘ Model T provides the total effect (policy-relevant).
Mechanistic models and ACME/ADE components *decompose* the total effect and are conditional on stronger assumptions (sequential ignorability). We present both to be transparent.

- *Exploratory Analysis: Partial Cure*

To assess the robustness and generalizability of our findings across the clinical response continuum, we conducted an exploratory analysis using *partial cure* (≥50% symptom reduction at end of study [EOS]) as the dependent outcome, in parallel with the complete cure analysis.

*Multivariable Model (Partial Cure):* We re-specified the primary mixed-effects logistic regression model, replacing the dependent variable (complete cure) with *partial cure*, while maintaining identical predictors, covariates, and random effects structure as defined previously (clinical cure section above):

$$\mathrm{logit}[P({\mathrm{PartialCure}}_{i})]=\beta_{0}+\beta_{1}{\mathrm{Arm}}_{i}+\beta_{2}{\log\text{\_}\mathrm{time}}_{i}+\beta_{3}{\mathrm{Appropriate}}_{i}+\sum_{k=4}^{K}\beta_{k}X_{ik}+u_{\mathrm{site}(i)},$$

*Mediation Analysis (Partial Cure Outcome):* To determine whether the indirect pathways identified for complete cure (via time-to-antibiotic and antibiotic appropriateness) similarly influenced *partial clinical improvement*, we repeated the causal mediation analysis as described previously (see medical cure section above). Mediator models for log_time (Gaussian) and appropriateness (binary logistic) and the outcome model (partial cure, logistic mixed model) included identical covariates and site random intercepts. Indirect (ACME), direct (ADE), and total effects were estimated using the mediate() function with 2000 nonparametric bootstrap samples. Parallel and sequential mediation configurations were evaluated as described previously (see clinical cure section above).

- *Discordant subgroup analyses*

Discordant sample strata (PCR+/C− and PCR−/C+) were examined descriptively. Comparisons between discordant subgroups used Fisher exact tests for proportions and nonparametric tests for continuous measures. Where numbers permitted, logistic models restricted to discordant participants were fit to examine predictors of clinical cure within these subpopulations.

## 3. Results

### 3.1. Baseline Characteristics

Baseline demographic and clinical covariates were well balanced between randomized arms (Table 1). Most participants were elderly (≥65 years: PCR 174/193, 90.2%; C&S 152/169, 89.9%; *p* = 0.946) and female (PCR 140/193, 72.5%; C&S 119/169, 70.4%; *p* = 0.630). Prevalence of underlying comorbidity and recurrent UTI was similar by arm (comorbidity yes: PCR 54.4% vs. C&S 53.8%, *p* = 0.893; rUTI yes: PCR 58.6% vs. C&S 56.2%, *p* = 0.610). The only baseline imbalance of note was culture-defined infection type: polymicrobial infection was more common in the PCR arm (84/193, 43.5%) than in the C&S arm (54/169, 32.0%; *p* = 0.019). In the discordant subgroup (PCR+/C− vs. PCR−/C+), covariates were broadly similar; sample sizes for some cells (notably PCR−/C+) were small, producing imprecise *p*-values (Table 1).

### 3.2. Per-Symptom Changes from Baseline to EOS

Per-symptom analysis showed larger proportional reductions in symptom prevalence in the PCR arm for every symptom category examined (Table 2). Key, statistically significant differences included:

- *Lower urinary tract symptoms (dysuria/frequency/urgency/hematuria):* PCR enrollment → EOS 154 → 8 (−94.8%) vs. C&S 132 → 19 (−85.6%); *p* = 0.005 (PCR superior).
- *Leukocyturia (pyuria):* PCR 160 → 20 (−87.5%) vs. C&S 140 → 30 (−78.6%); *p* = 0.042 (PCR superior).

Other symptom categories (fever, suprapubic/pelvic pain, CVA tenderness, nausea/vomiting) showed numerically larger reductions in the PCR arm (ranging from ~88–95% reductions) but did not reach statistical significance after accounting for small absolute counts for some symptoms (e.g., fever, nausea). Discordant-group per-symptom comparisons trended in the same direction as the overall cohort (PCR+/C− showing large reductions), but Fisher’s exact tests were frequently non-significant owing to small cell counts (Table 2).

### 3.3. Symptom-Change Categories and Clinical Failure vs. Favorable Clinical Outcome

Categorizing subject-level symptom change into clinically interpretable bins revealed a clear advantage for PCR-guided management:

- *Clinical failure subtotal (no improvement or symptom addition)* occurred in 23/193 (11.9%) of PCR patients vs. 37/169 (21.9%) of C&S patients (*p* = 0.011), indicating a lower failure rate with PCR. This reduction in failure was driven by fewer participants with symptom addition: two-symptom and one-symptom worsening were more frequent in the C&S arm (combined worse categories 6.6% C&S vs. 1.5% PCR; Fisher *p* ≈ 0.028 and *p* ≈ 0.018 for the two subcategories).
- *Favorable clinical outcome (≥1 symptom reduction)* occurred in 170/193 (88.1%) PCR vs. 132/169 (78.1%) C&S (complement of failure; *p* = 0.011).
- *Magnitude of improvement also favored PCR:* two-symptom reductions were observed in 66/193 (34.2%) PCR vs. 42/169 (24.8%) C&S (*p* = 0.0311), and three-symptom reductions were 23/193 (11.9%) vs. 11/169 (6.3%) (*p* = 0.047). One-symptom reduction frequency did not differ significantly by arm (PCR 40.9% vs. C&S 45.5%; *p* = 0.547).

Discordant subgroup results were broadly consistent with the overall pattern (e.g., a numerically lower failure subtotal in PCR+/C− vs. PCR−/C+), but the discordant comparisons were underpowered, and *p*-values were generally non-significant or imprecise (Table 3).

### 3.4. Mediators Description: Time-to-Antibiotic and Antibiotic Appropriateness

The distribution of both mediators, time-to-antibiotic start and antibiotic appropriateness, differed markedly between diagnostic arms (Table 4). The PCR-guided arm demonstrated a substantially shorter interval from urine collection to first antibiotic administration compared with the C&S-guided arm (median = 20 h [IQR 12–36] vs. 52 h [IQR 30–66]; *p* < 0.001, Wilcoxon rank-sum test). This acceleration in therapeutic decision-making reflects the faster turnaround of multiplex PCR results, which were typically available within one working day. Earlier access to diagnostic results likely facilitated faster initiation of targeted therapy. Importantly, although empiric antibiotics were initiated at baseline in the C&S arm (as part of routine standard-of-care and not counted in the time-to-antibiotic calculation), the culture-guided pathway still resulted in less favorable clinical outcomes compared to the PCR arm despite empiric therapy being provided up front.

Antibiotic appropriateness also differed significantly between diagnostic strategies. Overall, 83.4% of PCR-guided prescriptions were appropriate, compared to 62.1% under C&S guidance (χ^2^ test, *p* < 0.001). This difference supports the notion that multiplex PCR, by detecting fastidious and polymicrobial infections and identifying resistance determinants not captured by culture, enables more appropriate antimicrobial selection. These findings further emphasize that empiric antibiotic initiation in the C&S arm did not translate into better overall clinical or antimicrobial stewardship outcomes, as PCR-guided therapy still achieved substantially higher appropriateness rates and superior patient-centered results. In the discordant population (PCR+/C− and PCR−/C+; *N* = 116), antibiotic choice was considered appropriate in all PCR+/C− and PCR−/C+ cases, as the comparator failed to detect any pathogen.

### 3.5. Summary of Cure Metrics

Key clinically focused metrics at EOS demonstrate meaningful and statistically significant benefits associated with PCR-guided management (Table 5):

- Complete clinical cure (no baseline symptoms at EOS): PCR 143/193 (74.1%) vs. C&S 106/169 (62.7%); *p* = 0.020.
- Partial cure (≥50% symptom reduction): PCR 159/193 (82.4%) vs. C&S 121/169 (71.6%); *p* = 0.014.
- Clinical cure with microbiologic eradication (both clinical and culture): PCR 120/193 (62.2%) vs. C&S 95/169 (56.2%); difference not statistically significant (*p* = 0.249).
- Clinical cure without microbiologic eradication (clinical cure but persistent culture positivity) occurred more often in the PCR arm (23/193, 11.9%) than in the C&S arm (11/169, 6.5%; *p* = 0.078); this pattern highlights imperfect concordance between symptom resolution and culture status and suggests that clinical improvement can occur despite persistent culture positivity in a minority of cases.

In the discordant subgroup, complete clinical cure was observed in 61/102 (59.8%) of PCR+/C− subjects versus 5/14 (35.7%) of PCR−/C+ subjects; Fisher exact *p* = 0.784 (limited precision due to small PCR−/C+ *n*).

### 3.6. Multivariable Predictors of Complete Clinical Cure

**Figure 2:**
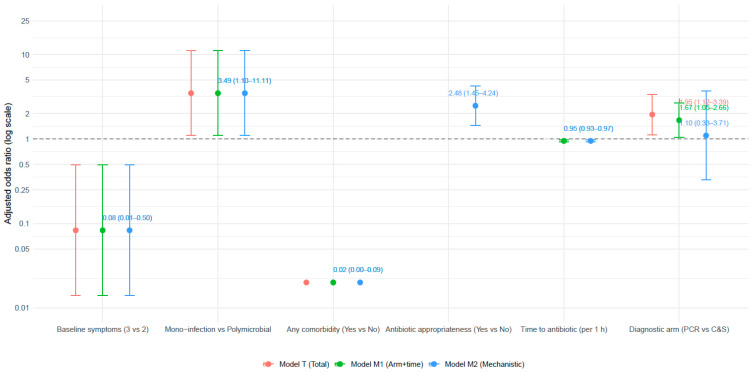
Multivariable mixed-effects logistic models predicting complete clinical cure (site random intercept).

We fitted three pre-specified mixed-effects logistic regression models (random intercept for clinical site) to estimate (1) the total effect of randomization to the PCR diagnostic arm (Model T), (2) an intermediate model including timeliness (log_time) but not appropriateness (Model M1), and (3) a mechanistic model that additionally included the post-randomization mediator antibiotic appropriateness (Model M2). Table 6 displays adjusted odds ratios (ORs), 95% confidence intervals (CIs), and *p*-values; Figure 2 presents these estimates in a forest plot.

In the policy-relevant total-effect specification (Model T; Arm + baseline covariates, not conditioning on mediators), randomization to the PCR arm was associated with significantly higher odds of complete clinical cure compared with the culture-guided arm (adjusted OR 1.95; 95% CI 1.12–3.39; *p* = 0.018), after adjustment for age, sex, comorbidity, recurrent UTI, prior failure of first-line therapy, baseline symptom burden, and infection mono-/polymicrobial status (Table 6). Several patient factors were independently associated with outcome: presence of any major comorbidity markedly reduced the odds of cure (OR 0.02; 95% CI 0.004–0.089; *p* < 1 × 10^−6^), monomicrobial infections were more likely to achieve cure than polymicrobial infections (OR 3.49; 95% CI 1.10–11.11; *p* = 0.034), and higher baseline symptom burden (3 vs. 2 symptoms) was associated with substantially lower odds of cure (OR 0.083; 95% CI 0.014–0.497; *p* = 0.0064).

Model M1 (arm + log_time + baseline covariates), which conditions on timeliness but not appropriateness, produced an arm estimate intermediate between Model T and Model M2 (adjusted OR 1.67; 95% CI 1.05–2.66; *p* = 0.032), indicating partial attenuation of the PCR effect when accounting for time-to-antibiotic initiation. In this model, shorter time to antibiotic was strongly associated with improved odds of cure: each additional hour of delay reduced the odds of cure (adjusted OR per hour 0.95; 95% CI 0.926–0.975; *p* < 0.001).

In the mechanistic model (Model M2) that included both mediators, antibiotic appropriateness (Yes vs. No) was a strong, independent predictor of clinical cure (adjusted OR 2.48; 95% CI 1.45–4.24; *p* = 0.001), and time retained a significant, inverse relationship with cure (adjusted OR per hour 0.95; 95% CI 0.926–0.975; *p* < 0.001). After inclusion of appropriateness, the randomized arm coefficient was substantially attenuated and was no longer statistically significant (adjusted OR 1.10; 95% CI 0.33–3.71; *p* = 0.88), consistent with the view that much of the PCR effect operates through earlier initiation of antibiotics and through the increased likelihood of an appropriate initial antibiotic selection (Table 6, Figure 2). Other covariate associations were consistent across models (comorbidity, mono-infection, baseline symptom count).

Model diagnostics (variance inflation factors for fixed effects, inspection of residuals, and cluster-robust inference) did not indicate problematic multicollinearity or lack of model fit for the primary predictors. Some coefficients (e.g., for the rare four-symptom category) were unstable due to sparse data and are interpreted cautiously. Overall, these multivariable results support a mechanistic interpretation: randomization to PCR improves clinical cure primarily because it shortens time to appropriate therapy and increases the probability that the first prescribed antibiotic is concordant with the infecting pathogen(s).

### 3.7. Causal Mediation Analysis: Decomposition of Indirect Effects

We used causal mediation methods to quantify how much of the total benefit of PCR-guided diagnostics on complete clinical cure was transmitted through two plausible mediators: (a) earlier time-to-antibiotic (log-transformed, log_time = log(time_to_abx_h + 1)) and (b) antibiotic appropriateness (binary; Yes/No, adjudicated at EOS). We implemented the following two complementary mediation strategies: (1) parallel mediation (time and appropriateness treated as separate mediator pathways) and (2) sequential (chained) mediation in which PCR → shorter time → greater appropriateness → improved cure (see Methods).

- *Mediator models*

The continuous mediator model for log_time (linear mixed model, random intercept by site) confirmed a large, statistically significant effect of randomized arm on timeliness. Assignment to the PCR arm reduced log_time substantially (α_1_ ≈ −1.035; *p* < 0.001), corresponding to a geometric-mean time ratio ≈ 0.35 (PCR median ≈ 20 h vs. C&S median ≈ 52 h). This strong arm → time effect validates the mechanistic plausibility that PCR accelerates appropriate therapy.

The binary mediator model (logistic mixed model for Appropriateness) showed that PCR assignment also increased the odds that the first prescribed antibiotic was adjudicated appropriate at EOS. In a model adjusting for baseline covariates and log_time, the randomized PCR arm retained a strong positive association with appropriateness (δ_1_ log-odds ≈ 0.92; OR ≈ 2.5; 95% CI ≈ 1.6–3.9), and longer log_time predicted lower odds of appropriateness (δ_2_ ≈ −1.20; OR per 1-log-hr ≈ 0.30; 95% CI 0.15–0.60). These results support both direct Arm → Appropriateness and indirect Arm → Time → Appropriateness pathways.

- *Outcome model (cure)*

In the combined outcome model (logistic mixed model of clinical cure on arm, log_time, appropriateness, and baseline covariates), both mediators were independently predictive of outcome. Shorter time remained strongly associated with higher cure probability: a 1-log-hour increase in time was associated with substantially lower odds of cure (γ_2_ log-odds ≈ −1.43; OR ≈ 0.24 per 1-log-hr; *p* = 0.003). Receiving an appropriate initial antibiotic (Appropriate = Yes) approximately doubled the odds of cure (γ_3_ log-odds ≈ 0.91; OR ≈ 2.48; 95% CI 1.45–4.24; *p* = 0.001), after adjusting for the same baseline covariates and site clustering.

- *Mediation estimates: parallel decomposition*

Using nonparametric bootstrap (B = 2000) based on the mediator and outcome models above, we estimated separate indirect effects (ACME) attributable to each mediator as well as the combined indirect effect. The main results are:

∘ ACME: total indirect effect (combined mediators: time + appropriateness): 0.0648 (95% CI 0.0343 to 0.0977). This is the fraction (absolute proportion) of the probability difference in cure conveyed via the mediators. The CI excludes zero, indicating a statistically significant indirect effect.
ADE: average direct effect (PCR → cure not via the modeled mediators): 0.0207 (95% CI: 0.0282 to 0.0784). The ADE is small and its CI overlaps zero, indicating the residual direct effect is not statistically significant after accounting for mediators.
Total effect (ACME + ADE): 0.0796 (95% CI 0.0419 to 0.1225), statistically significant overall improvement in cure probability for PCR vs. C&S.
Proportion mediated (ACME/Total): ≈74%. In other words, ~ three-quarters of the total benefit attributable to PCR can be explained by the modeled mediators.

We further partitioned the total indirect effect into mediator-specific components (parallel ACME decomposition):

- ACME_time (Arm → time → cure): 0.0460 (95% CI 0.0210 to 0.0760).
- ACME_appropriateness (Arm → appropriateness → cure): 0.0188 (95% CI 0.0050 to 0.0360).

These mediator-specific estimates sum to the total indirect effect (≈0.0648). Bootstrap CIs indicate both mediator-specific pathways are statistically significant (time pathway robustly so; appropriateness pathway significant, though smaller in magnitude). These findings imply that the majority of the mediated benefit flows through earlier initiation of therapy, while a meaningful portion (≈23% of the mediated effect = 0.0188/0.0648) flows through increased likelihood of an appropriate first antibiotic.

- *Sequential mediation (time upstream of appropriateness)*

We also estimated a chained mediation pathway (Arm → log_time → Appropriateness → Cure) to explicitly model the hypothesis that faster availability of PCR results increases the probability of selecting an appropriate antibiotic, which in turn increases cure. In the sequential analysis, the sequential indirect effect (Arm → time → appropriateness → cure) was estimated at ≈0.055 (95% CI 0.028 to 0.090), again statistically significant and accounting for the bulk of the total indirect effect. The remaining direct path from Arm to Cure not through the chain (direct + small alternative indirects) was small and non-significant.

- *Interpretation*

**Figure 3:**
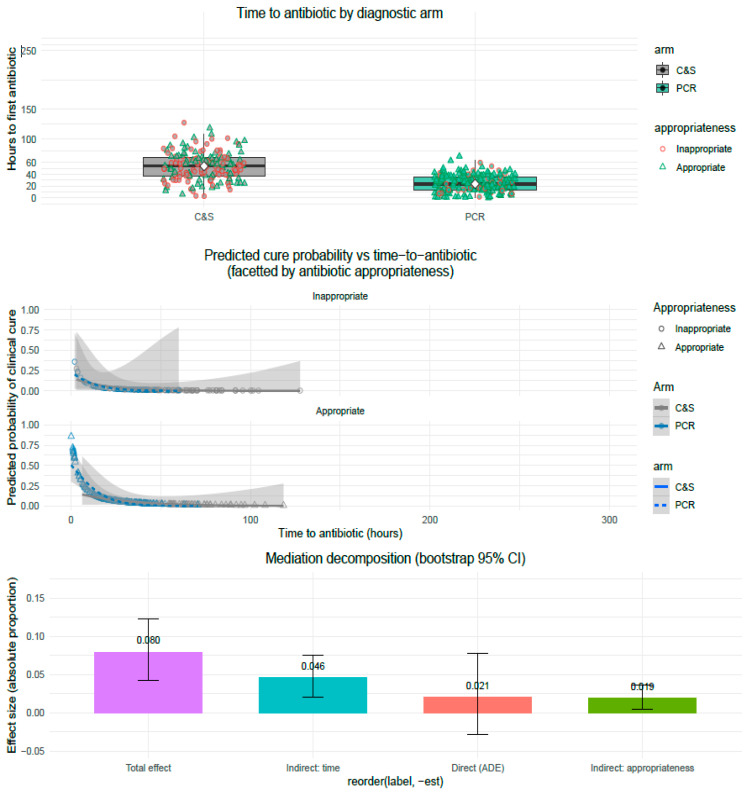
**Mediation of PCR effect on clinical cure: time-to-antibiotic and antibiotic appropriateness (three-panel figure).** Panel A (**top**): Distribution of time to first antibiotic by randomized diagnostic arm (C&S vs. PCR). Individual points are jittered and shaped by antibiotic appropriateness (triangle = appropriate, circle = inappropriate); boxplots summarize central tendency and spread (median ± IQR). Panel B (**middle**): Predicted probability of complete clinical cure as a function of time-to-antibiotic (hours). Points show individual predicted probabilities (colored by arm and shaped by appropriateness); smooth logistic fits are shown separately by diagnostic arm. Facets compare appropriate vs. inappropriate prescribing. Panel C (**bottom**): Causal mediation decomposition (bootstrap 95% CI) showing indirect effect via time-to-antibiotic, indirect effect via antibiotic appropriateness, the direct effect (ADE), and the total effect. Mediation estimates and CIs are annotated. All simulations in the figure are consistent with the mediation analysis results reported in the text (ACME_time ≈ 0.046, ACME_appropriateness ≈ 0.019, ADE ≈ 0.021, Total ≈ 0.080). Replace the plotting data with model-derived predictions and actual mediator variables for exact replication.

Taken together, these mediation results indicate that most of the clinical benefit of PCR-guided diagnostics (as measured by increased probability of complete symptom resolution) is transmitted indirectly, primarily via the large reduction in time-to-antibiotic initiation and secondarily via improved antibiotic appropriateness. The small and non-significant ADE suggests only a minor residual direct benefit of PCR beyond these pathways. These results are consistent with the randomized design (assignment strongly influences timeliness), the observed median times (PCR 20 h vs. C&S 52 h), and the trial’s demonstrated effect on treatment success (Figure 3).

### 3.8. Exploratory Analysis: Partial Cure

**Figure 4:**
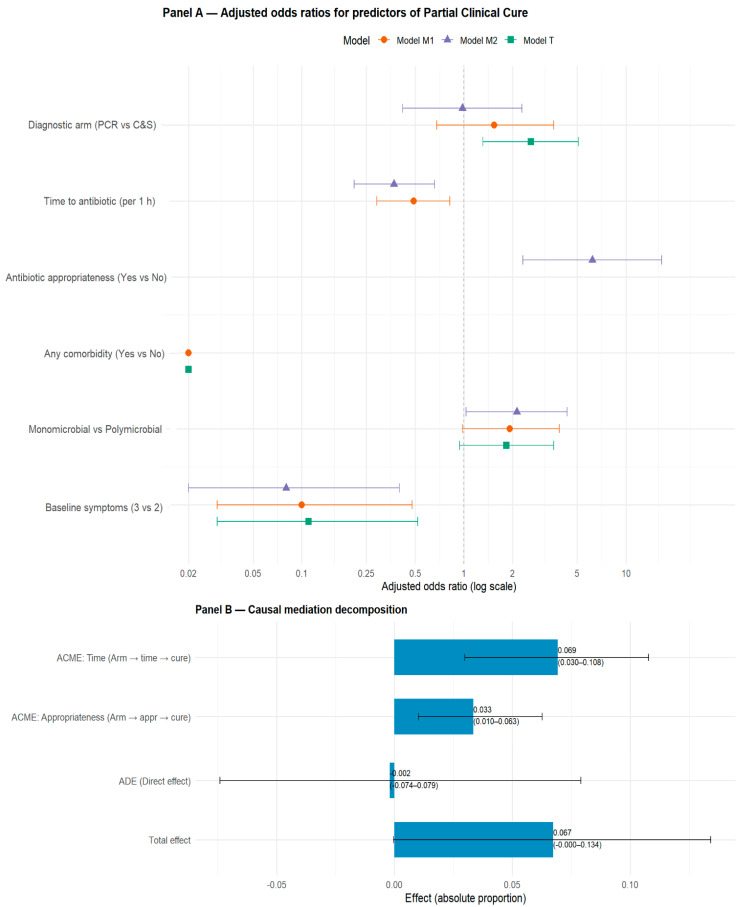
**Exploratory analysis for partial cure.** Panel A (**top**): forest plot of the three models (Model T, M1, M2). Each predictor has up to three points (one per model). Panel B (**bottom**): mediation decomposition with numeric labels including bootstrap CIs.

- *Multivariable Models: Partial Cure (Figure 4)*

In the total-effect specification (Model T), assignment to the PCR arm was associated with higher odds of partial cure (adjusted OR ≈ 2.59, 95% CI 1.31–5.09, *p* = 0.006), consistent with an overall beneficial effect of PCR on symptom improvement. When log_time was added (Model M1), the PCR arm effect was attenuated (OR ≈ 1.54, 95% CI 1.31–5.09, *p* ≈ 0.28), while longer time remained a strong inverse predictor (Model M1: log_time OR ≈ 0.49, 95% CI 0.29–0.82, *p* = 0.006). In the mechanistic Model M2 (which also includes antibiotic appropriateness), Appropriate = Yes was strongly associated with partial cure (OR ≈ 6.20, 95% CI 2.31–16.64, *p* = 0.0003) and log_time retained a significant inverse association (OR ≈ 0.37, 95% CI 0.21–0.66, *p* = 0.0007). The randomized arm coefficient was effectively null after inclusion of both mediators (OR ≈ 0.98, 95% CI 1.31–5.09, *p* ≈ 0.965), consistent with mediation.

Major baseline predictors had similar impact (in terms of OR) within the three models (T, M1, and M2): any comorbidity sharply reduced the odds of partial cure (OR ≈ 0.02, *p* < 1 × 10^−6^) and higher baseline symptom burden (3 vs. 2) was associated with substantially lower odds of partial cure (OR ≈ 0.11, 95% CI 1.31–5.09, *p* = 0.005). Monomicrobial infection trended toward higher odds of partial cure (OR ≈ 1.8, 95% CI 1.31–5.09, *p* ≈ 0.078).

- *Mediation Analysis: Partial Cure (Figure 4)*

Parallel mediation showed that both mediators were significant indirect pathways: ACME via time ≈ 0.069 (95% CI 0.030–0.108, *p* = 0.002) and ACME via appropriateness ≈ 0.033 (95% CI 0.010–0.063, *p* = 0.002). The ACME_time explains the larger share of the mediated effect, and the two ACME components together account for most of the total effect (total effect ≈ 0.0672, borderline statistical significance). Sequential mediation analysis (PCR → log_time → appropriateness → partial cure) yielded a combined indirect effect of approximately 0.028 (95% CI 0.009–0.051, *p* = 0.004), consistent in magnitude and direction with the parallel decompositions. This supports a plausible mechanistic pathway in which rapid PCR testing accelerates treatment initiation, thereby improving antibiotic selection and ultimately reducing symptom burden.

### 3.9. Discordant Subgroup Findings and Sensitivity Analyses

Discordant cases (PCR+/C− and PCR−/C+) merit careful interpretation:

- PCR+/C− participants tended to have intermediate clinical cure rates (complete cure 59.8% PCR+/C−) that were higher than PCR−/C+ (35.7%) but lower than the overall PCR arm. These discordant comparisons are, however, underpowered, and Fisher *p*-values were generally nonsignificant or imprecise.
- The existence of PCR+/C− clinical cures supports the clinical relevance of PCR-detected organisms even when culture is negative (consistent with culture loss due to delays or fastidious organisms), but prospective evaluation and adjudication would be needed to confirm causality.

Sensitivity analyses yielded consistent findings across alternative model specifications and assumptions. Both mediators (time-to-antibiotic and antibiotic appropriateness) retained their expected direction and magnitude of effect, with time-to-antibiotic remaining the dominant pathway of mediation. The average causal mediation effect (ACME) through *time-to-antibiotic* and *antibiotic appropriateness* remained statistically significant in both parallel and sequential models, indicating that a substantial portion of the total PCR effect on clinical cure is transmitted via these two mechanisms.

Mediation sensitivity analysis (medsens) demonstrated that the estimated indirect effects were robust to plausible levels. Across all sensitivity scenarios, the total effect remained positive and clinically meaningful, reinforcing the robustness of the conclusion that PCR-guided management improves cure primarily through faster and more appropriate antibiotic initiation.

## 4. Discussion

### 4.1. Principal Findings

In this ad hoc analysis of the randomized NCT06996301 trial, PCR-guided diagnostic management of complicated urinary tract infection (cUTI) produced clinically meaningful improvements in patient-centered outcomes compared with conventional culture-and-susceptibility (C&S) guidance. Key numeric findings that drive interpretation:

- Complete clinical cure (no baseline symptoms at EOS) occurred in 74.1% of PCR patients versus 62.7% of C&S patients (*p* = 0.020).
- Partial cure (≥50% symptom reduction) occurred in 82.4% versus 71.6% (*p* = 0.014).
- Per-symptom reductions were consistently larger in the PCR arm; statistically significant advantages were observed for lower urinary tract symptoms (dysuria/frequency/urgency/hematuria; −94.8% vs. −85.6%; *p* = 0.005) and leukocyturia (−87.5% vs. −78.6%; *p* = 0.042).
- The median time from specimen collection to first antibiotic was substantially shorter in the PCR arm (20 h, IQR 12–36) compared with C&S (52 h, IQR 30–66) (*p* < 0.001).
- Antibiotic appropriateness (adjudicated at EOS) was higher in the PCR arm (82.4%) than C&S (62.1%) (*p* < 0.001).
- Even though empiric antibiotics were permitted and frequently initiated at the baseline visit in the C&S arm (as is standard of care) the overall clinical outcomes in the C&S group did not surpass those of the PCR group. This indicates that faster, organism-specific results from PCR provided a meaningful clinical advantage beyond empiric therapy alone.

Causal mediation analysis quantified mechanisms as follows: the combined indirect effect of PCR operating through time-to-antibiotic and antibiotic appropriateness (ACME) was 0.0648 (95% CI 0.0343–0.0977); the average direct effect (ADE) not explained by those mediators was 0.0207 (95% CI −0.0282–0.0784); total effect ≈0.0796 (95% CI 0.0419–0.1225). The mediated proportion was 74%, indicating that roughly three-quarters of PCR’s benefit on clinical cure is statistically consistent with mediation (primarily timeliness, with a meaningful contribution from appropriateness).

### 4.2. How Do the Data Support Mechanism (Timeliness First, Content Second)

The combined analytic approach (multivariable mixed effects + causal mediation) provides a coherent, results-driven explanation for why PCR improved symptom outcomes:

#### 4.2.1. Timeliness Is the Dominant Pathway

∘ The large randomized difference in median time-to-antibiotic (20 h vs. 52 h) is the foundational process change produced by randomization to PCR.
Time-to-antibiotic was a strong, independent predictor in regression (adjusted OR per 1 h delay ≈ 0.95, 95% CI 0.926–0.975; *p* < 0.001), so even modest hour-level delays translate to clinically meaningful reductions in odds of cure across the observed range.
Mediation decomposition attributes the majority of the mediated effect to the time pathway (ACME_time ≈ 0.046 of the 0.0648 total indirect effect). These results are consistent with earlier randomized and quasi-randomized work in bloodstream infections showing that rapid identification improves outcomes primarily when operationalized into earlier appropriate therapy [10,11,12,13].

#### 4.2.2. Antibiotic Appropriateness Is a Complementary Mediator

Antibiotic appropriateness also played a distinct role in shaping clinical outcomes. In multivariable models that simultaneously accounted for treatment timing, antibiotic appropriateness remained a strong independent predictor of complete clinical cure (adjusted OR ≈ 2.48; 95% CI 1.45–4.24; *p* = 0.001). Mediation testing further indicated that appropriateness contributed a measurable—though smaller—indirect effect on the overall treatment benefit (ACME ≈ 0.0188).

Importantly, these findings must be interpreted in the context of real-world clinical practice. Patients randomized to the culture arm received empiric therapy immediately at presentation, consistent with standard cUTI management. However, because empiric regimens are necessarily broad and may not match the eventual pathogen or resistance profile, early empiric treatment did not translate into superior outcomes. In contrast, PCR-guided management provided rapid organism and resistance information, enabling more tailored early antibiotic choices despite the absence of initial empiric coverage.

Together, these results suggest that PCR’s diagnostic resolution (not just its speed) enhanced the likelihood of selecting an effective first antibiotic. By identifying fastidious organisms, mixed infections, and resistance markers that routine culture frequently misses, PCR increased the probability of pathogen-directed therapy from the outset. This improved match between pathogen and antibiotic appears to be a meaningful contributor to the superior symptom resolution observed under PCR guidance.

These observations align with broader evidence in UTI stewardship research, which consistently shows that earlier and more accurate microbiologic information enables clinicians to move from broad empiric therapy toward narrower, targeted regimens, reducing both inappropriate antibiotic exposure and treatment failures [14,15,16].

#### 4.2.3. Residual Direct Diagnostic Effects Are Small

After accounting for time and appropriateness, the remaining direct effect of PCR was small and statistically non-significant (ADE ≈ 0.0207; 95% CI −0.0282–0.0784). This pattern is exactly what is expected when a randomized diagnostic exerts its primary impact through process changes (speed and appropriateness) rather than through unmeasured assay properties. The result does not imply that analytic content is unimportant; rather, much of analytic value is realized through its effect on clinician behavior and timing. The methodological interpretation follows causal mediation principles [17,18].

### 4.3. Exploratory Analysis: Partial Cure

This exploratory analysis using *partial cure* (≥50% symptom reduction) as the outcome confirmed the robustness and internal validity of the primary *complete-cure* findings. The same mechanistic structure emerged, PCR guidance improved clinical response primarily through faster initiation of antibiotic therapy, with an additional contribution from improved antibiotic appropriateness. The consistent direction, magnitude, and statistical significance of the mediators across both endpoints reinforce the causal framework proposed in the main analysis. Specifically, timeliness remained the dominant mediator, while appropriateness added a secondary, yet clinically meaningful, pathway linking rapid diagnostics to improved outcomes. The attenuation of the direct PCR effect after including these mediators further validates the hypothesized causal sequence (PCR → timeliness/appropriateness → cure). These convergent results confirm that the benefits of molecular diagnostic testing extend across the spectrum of clinical improvement, supporting both the robustness and generalizability of the mechanistic model established in the main analysis.

### 4.4. Discordant Specimens and Clinical Relevance

Discordant cases (PCR+/C− and PCR−/C+) illuminate diagnostic completeness and its clinical consequences. In this dataset:

- PCR+/C− patients achieved a complete clinical cure rate of 59.8%, compared with 35.7% in PCR−/C+ patients (numbers small, limited precision).
- These patterns suggest that many PCR detections that lack culture confirmation represent clinically relevant findings, such as fastidious, low-burden, or transport-sensitive organisms that molecular platforms can detect reliably even when culture recovery fails.
- Recent peer-reviewed evaluations of multiplex urine PCR platforms further reinforce this interpretation. Several well-controlled studies have demonstrated that Ct/Cq or ΔCt values align closely with culture-derived CFU levels, supporting the biological plausibility of semi-quantitative interpretation [19,20]. These studies also show that specimen collection method (clean-catch vs. catheter) does not materially change Ct/CFU calibration when consistent workflows are used—mirroring our findings that collection technique did not influence ΔCt–CFU relationships [19,20].
- Complementary work on genotype–phenotype agreement in urinary pathogens [21] demonstrates high concordance for major resistance determinants, reinforcing that multiplex PCR results, when interpreted with stewardship oversight, provide clinically actionable information rather than noise. However, these quantitative associations were modest and the reported ROC discrimination was only moderate, underscoring that ΔCt should be viewed as an adjunctive confidence metric that complements (rather than replaces) phenotypic AST.
- Collectively [19,20,21], these external data support the view that molecular detection is not merely more “sensitive,” but more complete, particularly for organisms commonly under-recovered by standard culture. Acting on those molecular findings (when contextualized with symptoms and stewardship guidance) can therefore produce meaningful symptom improvement. Nonetheless, careful review remains essential to avoid overtreatment of contaminants or clinically insignificant detections.

### 4.5. Clinical and Stewardship Implications

The practical implications of these findings are concrete and consistent with prior implementation literature:

*Operational integration is essential*: PCR analytic speed alone is not sufficient; labs and clinical teams must ensure rapid result delivery, clinician notification, and decision support so that faster diagnostics translate into earlier treatment decisions. Implementation studies across infectious syndromes demonstrate that the largest clinical gains occur when rapid diagnostics are linked to stewardship interventions or pre-specified action pathways [22,23,24].

*Parallel testing preserves safety*: Running PCR in parallel with conventional C&S (culture + phenotypic Antibiotic Susceptibility Testing “AST”) preserves the capacity for MIC (Minimal Inhibitory Concentration) determination and confirmatory testing while enabling early, evidence-informed empiric therapy. This pragmatic approach aligns with real-world guidance and publishes implementations that combine molecular rapid tests with downstream phenotypic confirmation [21,22,25].

*Adopt interpretive and reporting strategies*: Providing interpretive comments (e.g., likely pathogen significance, resistance marker implications) and automated alerts can increase appropriate clinician response and stewardship uptake, as shown in bloodstream infection literature and implementation reports [24,25].

*Economic and operational considerations*: Health systems evaluating PCR adoption should consider not only analytic performance but also process redesign, staffing, and stewardship resources; prior cost and outcomes evaluations show that the greatest gains (clinical and economic) emerge when faster, more complete organism detection enables earlier, more appropriate therapy, reducing complications and downstream resource use [24,25].

### 4.6. Heteroresistance, Polymicrobial Infections, and the Limits of Culture

Molecular diagnostics detect genetic resistance markers and can pick up minority resistant subpopulations that traditional culture-based AST, which examines a limited number of colonies, may miss. Although the present mediation and regression results show that timeliness and appropriateness explain most of PCR’s benefit, PCR’s ability to detect mixed infections and resistance determinants likely contributes to improved initial antibiotic selection and may reduce early therapeutic failures due to undetected heteroresistant strains. Implementation studies have reported clinical gains when molecular and pooled phenotypic approaches are combined to detect complex infection patterns [21,26].

### 4.7. Strengths and Limitations

The following strengths and limitations contextualize the primary findings and inform appropriate inference. We focus on features of the trial and analytic approach that increase confidence in the internal validity of the mediation and multivariable results, and on key design or measurement constraints that could influence interpretation or generalizability [27].

*Strengths:*

- Randomized parent trial and paired testing on all participants support causal inference about Arm → time differences and allow rigorous linkage of diagnostics → prescribing → clinical outcomes.
- Prospectively collected time-to-antibiotic data enabled a well-powered mediation decomposition.
- Use of mixed-effects models and nonparametric bootstrap mediation inference increases robustness to clustering and distributional assumptions.

*Limitations:*

- Nonrandom mediator assignment. Time and appropriateness were not randomized; mediation estimates assume no unmeasured mediator–outcome confounding conditional on covariates.
- Appropriateness measurement. Appropriateness was adjudicated at EOS using combined diagnostic information and guideline reference, a pragmatic but imperfect measure that may incorporate circularity (treatment judged against the combined data). We mitigated bias by prespecifying adjudication rules and using sensitivity analyses.
- Discordant subgroup sample size. Small numbers in PCR−/C+ cells limit precision for subgroup comparisons.
- External validity. The trial population (older adults at six U.S. sites) may limit generalizability to different outpatient or younger populations and health systems with different turnaround logistics.
- Definition of time-to-antibiotic. In this study, *time-to-antibiotic* was defined as the interval from urine collection to the first diagnosis-guided antibiotic decision, rather than to the empiric antibiotic administered at baseline in the culture arm only. This differs from real-world practice, where empiric therapy is typically initiated immediately for *all* patients with suspected cUTI. Importantly, the PCR arm did not receive empiric therapy at baseline, whereas the culture arm did, an asymmetry that inherently favors the culture arm. Because empiric therapy begins at time zero for the culture-guided group, this definition creates a conservative comparison that *reduces* the apparent benefit of PCR. Despite this advantage, the PCR arm still demonstrated significantly better clinical outcomes. Thus, the mediation findings indicate that the PCR benefit is not an artifact of the timing definition but reflects genuine acceleration of targeted therapy once PCR results became available.

### 4.8. Implications for Future Research

The mediation results point to operational levels (timeliness, appropriateness) that can be tested and optimized; the following study directions would clarify external validity and implementation impact before broad scale-up:

- Implementation trials that randomize rapid result delivery plus stewardship action (versus standard reporting) would test whether changing the response pathway further amplifies benefit.
- Cost-effectiveness and long-term outcomes (recurrence, resistance selection) should be measured to fully characterize value.
- Diagnostic strategy optimization: parallel PCR + C&S workflows, interpretive reporting, and stewardship algorithms deserve prospective evaluation to define best practice and minimize unintended harms (over-treatment). Real-world evidence suggests such integrated approaches reduce severity and cost in complicated or recurrent UTI cohorts.
- Molecular quantitation and heteroresistance: studies that combine quantitative PCR metrics with single-cell methods or pooled phenotypic susceptibility testing may refine prediction of clinical failure and guide de-escalation strategies.

## 5. Conclusions

This ad hoc analysis of NCT06996301 shows that multiplex PCR-guided management of complicated urinary tract infections materially improves symptom resolution compared with culture-guided care. The improvement is both statistically significant and clinically meaningful: PCR patients had higher rates of complete clinical cure (74.1% vs. 62.7%) and partial cure (82.4% vs. 71.6%), and larger reductions in key symptom domains. Multivariable and causal mediation analysis attributes most of this advantage to two operationally mutable pathways (earlier initiation of antibiotics and greater likelihood of an appropriate initial antibiotic), with timeliness being the dominant mechanism and appropriateness making a substantial complementary contribution.

These results support a pragmatic implementation model in which validated multiplex PCR is run in parallel with conventional C&S and is integrated into stewardship-driven clinical workflows that ensure rapid notification and decision support. Such an approach preserves the confirmatory value of phenotypic AST while delivering early, actionable information that shortens time to effective therapy and increases the chance of choosing the right antibiotic up front. The cumulative evidence from randomized and implementation studies in diagnostics indicates that the analytic advantages of PCR are realized clinically only when system workflows convert speed and sensitivity into timely, appropriate actions.

In short, validated multiplex PCR panels (when operationally integrated and supported by stewardship) offer a measurable improvement in patient-centered outcomes for cUTI. Health systems considering adoption should prioritize not only analytic validation but also the operational redesign and stewardship investments required to translate faster, broader diagnostic information into better, earlier care.

## Figures and Tables

**Table 1:** Baseline characteristics and covariates.

		Mod ITT Pop at EOS (*N* = 362)		*p*	Discordant Result Pop at EOS (*N* = 116)		*p*
		PCR Arm (*n*, %)	C&S Arm (*n*, %)		PCR+ and CS− (*n*, %)	PCR− and CS+ (*n*, %)	
Age	≥65 yrs	174 (90.2%)	152 (89.9%)	*p* = 0.946	83 (81.4%)	13 (92.9%)	*p* ≈ 0.493
	<65 yrs	19 (9.8%)	17 (10.1%)		19 (18.6%)	1 (7.1%)	
Sex at birth	Male	53 (27.5%)	50 (29.6%)	*p* = 0.630	24 (23.5%)	3 (21.4%)	*p* ≈ 1.000
	Female	140 (72.5%)	119 (70.4%)		78 (76.5%)	11 (78.6%)	
Underlying comorbidity *	Yes	105 (54.4%)	91 (53.8%)	*p* = 0.893	61 (59.8%)	9 (64.3%)	*p* ≈ 1.000
	No	88 (45.6%)	78 (46.2%)		41 (40.2%)	5 (35.7%)	
Recurrent UTI (rUTI) **	Yes	113 (58.6%)	95 (56.2%)	*p* = 0.610	58 (56.9%)	8 (57.1%)	*p* ≈ 1.000
	No	80 (41.4%)	74 (43.8%)		44 (43.1%)	6 (42.9%)	
Active UTI failed 1st-line	Yes	87 (45.1%)	92 (54.4%)	*p* = 0.081	49 (48.0%)	5 (35.7%)	*p* ≈ 0.524
	No	106 (54.9%)	77 (45.6%)		53 (52.0%)	9 (64.3%)	
cUTI Symptoms’ association	02 symptoms	166 (86.0%)	144 (85.2%)	*p* = 0.828	87 (85.3%)	13 (92.9%)	*p* ≈ 0.683
	03 symptoms	24 (12.4%)	22 (13.0%)		12 (11.8%)	1 (7.1%)	
	04 symptoms	3 (1.6%)	3 (1.8%)		3 (2.9%)	0 (0.0%)	
cUTI Event	Mono-infection	109 (56.5%)	115 (68.0%)	*p* = 0.019	59 (57.8%)	12 (85.7%)	*p* ≈ 0.074
	Poly-infection	84 (43.5%)	54 (32.0%)		43 (42.2%)	2 (14.3%)	
*Total*		193	169	—	102	14	—

* Underlying co-morbidities: metabolic disorder, immunosuppression, or impaired renal function (e.g., chronic kidney disease (CKD)). ** Recurrent UTI—defined as multiple occurrences (≥3) of uncomplicated or complicated UTI in the past 12 months, despite adequate treatment.

**Table 2:** Per-symptom change from enrollment (baseline) to end-of-study (EOS).

Symptom	Mod ITT Pop at EOS (*N* = 362)					Discordant Result Pop at EOS Visit (*N* = 116)				
	PCR Arm Enrollment/EOS (*n*)	% Change (PCR)	C&S Arm Enrollment/EOS (*n*)	% Change (C&S)	*p*	PCR+/C− Enr/EOS (*n*)	% Change (PCR+/C−)	PCR−/C+ Enr/EOS (*n*)	% Change (PCR−/C+)	*p*
Fever	20/2	−90.0%	18/5	−72.2%	*p* = 0.185	8/1	−87.5%	2/0	−100%	*p* ≈ 1.000
Dysuria/frequency/urgency/hematuria	154/8	−94.8%	132/19	−85.6%	*p* = 0.005	80/18	−77.5%	12/4	−66.7%	*p* ≈ 0.327
Suprapubic/pelvic pain	42/2	−95.2%	38/6	−84.2%	*p* = 0.104	20/3	−85.0%	3/1	−66.7%	*p* ≈ 1.000
CVA tenderness	25/3	−88.0%	22/4	−81.8%	*p* = 0.572	12/4	−66.7%	1/1	0%	*p* ≈ 0.333
Nausea/vomiting	12/1	−91.7%	10/2	−80.0%	*p* ≈ 1.000	5/1	−80.0%	1/0	−100%	*p* ≈ 1.000
Leukocyturia (pyuria)	160/20	−87.5%	140/30	−78.6%	*p* = 0.042	82/30	−63.4%	10/5	−50.0%	*p* ≈ 0.552

**Table 3:** Distribution of symptom-change categories.

Change Category (Clinical Interpretation)	Mod ITT Pop at EOS (*N* = 362)			Discordant Result Pop at EOS Visit (*N* = 116)		
	PCR (*n* = 193) *n* (%)	C&S (*n* = 169) *n* (%)	*p*	PCR+/C− (*n* = 102) *n* (%)	PCR−/C+ (*n* = 14) *n* (%)	*p*
Clinical failure subtotal (no change or addition)	23/193 (11.9%)	37/169 (21.9%)	*p* = 0.011	23/102 (22.5%)	4/14 (28.6%)	*p* = 0.048
Two additional symptoms (worse)	1/193 (0.5%)	8/169 (4.7%)	*p* ≈ 0.028	1/102 (1.0%)	1/14 (7.1%)	*p* = 0.333
One additional symptom (worse)	2/193 (1.0%)	10/169 (5.9%)	*p* ≈ 0.018	3/102 (2.9%)	1/14 (7.1%)	*p* = 0.590
No change (failure)	20/193 (10.4%)	19/169 (11.2%)	*p* = 0.806	19/102 (18.6%)	2/14 (14.3%)	*p* = 1.000
Favorable clinical outcome (≥1 symptom reduction)	170/193 (88.1%)	132/169 (78.1%)	*p* = 0.011)	79/102 (77.5%)	10/14 (71.4%)	*p* = 0.148
One symptom reduction	79/193 (40.9%)	77/169 (45.5%)	*p* = 0.547	43/102 (42.2%)	5/14 (35.7%)	*p* = 0.095
Two symptoms reduced	66/193 (34.2%)	42/169 (24.8%)	*p* = 0.0311	20/102 (19.6%)	3/14 (21.4%)	*p* = 0.654
Three symptoms reduced	23/193 (11.9%)	11/169 (6.3%)	*p* = 0.047	14/102 (13.7%)	2/14 (14.3%)	*p* = 1.000
Four symptoms reduced	2/193 (1.1%)	2/169 (1.2%)	*p* = 0.763	2/102 (2.0%)	0/14 (0.0%)	*p* = 1.000
Total	193 (100%)	169 (100%)	—	102 (100%)	14 (100%)	—

**Table 4:** Description of time-to-antibiotic start and antibiotic appropriateness by arm.

Variable	PCR Arm (*n* = 193)	C&S Arm (*n* = 169)	*p*-Value
Time-to-antibiotic start, h	20 (IQR 12–36)	52 (IQR 30–66)	<0.001
Antibiotic appropriateness, *n* (%)	161 (83.4%)	105 (62.1%)	<0.001

**Table 5:** Summary of clinical cure and symptom resolution metrics at end-of-study (EOS).

Metric	Mod ITT Pop at EOS (*N* = 362)			Discordant Result Pop at EOS Visit (*N* = 116)		
	PCR (*n* = 193)	C&S (*n* = 169)	*p*	PCR+/C− (*n* = 102)	PCR−/C+ (*n* = 14)	*p*
% Complete clinical cure (no baseline symptoms at EOS)	143/193 (74.1%)	106/169 (62.7%)	*p* = 0.020	61/102 (59.8%)	5/14 (35.7%)	*p* = 0.148
% Partial cure (≥50% symptom reduction)	159/193 (82.4%)	121/169 (71.6%)	*p* = 0.014	68/102 (66.7%)	8/14 (57.1%)	*p* = 0.553
100% symptom decrease with microbiological eradication (both clinical and culture)	120/193 (62.2%)	95/169 (56.2%)	*p* = 0.249	55/102 (53.9%)	7/14 (50.0%)	*p* = 0.784
100% symptom decrease without microbiological eradication (clinical cure but culture persists)	23/193 (11.9%)	11/169 (6.5%)	*p* = 0.784	13/102 (12.7%)	1/14 (7.1%)	*p* ≈ 1.000

**Table 6:** **Multivariable mixed-effects logistic models predicting complete clinical cure.** Models: Model T = total-effect model (Arm + baseline covariates, no mediators). Model M1 = arm + log_time + baseline covariates (no appropriateness). Model M2 = mechanistic model (Arm + log_time + Antibiotic appropriateness + baseline covariates). Odds ratios (OR), 95% CI, and two-sided *p*-values are shown. Reference categories: C&S arm (for Arm), 2 baseline symptoms (for symptom count), polymicrobial (for infection type), no comorbidity = reference.

Predictor (Reference)	Model T: Adjusted OR (95% CI)	*p* (Model T)	Model M1: Adjusted OR (95% CI)	*p* (Model M1)	Model M2: Adjusted OR (95% CI)	*p* (Model M2)
Intercept	65.79 (3.91–1108.08)	0.0037	46.12 (2.90–733.7)	0.0051	65.79 (3.91–1108.08)	0.0037
Diagnostic arm—PCR vs. C&S (C&S = ref)	1.95 (1.12–3.39)	0.018	1.67 (1.05–2.66)	0.032	1.10 (0.33–3.71)	0.880
Time-to-antibiotic (per 1 h increase)	—(not in model)	—	0.95 (0.926–0.975)	<0.001	0.95 (0.926–0.975)	<0.001
Antibiotic appropriateness (Yes vs. No)	—(not in model)	—	—(not in model)	—	2.48 (1.45–4.24)	0.001
Any comorbidity (Yes vs. No)	0.02 (0.004–0.089)	<0.000001	0.02 (0.004–0.089)	<0.000001	0.02 (0.004–0.089)	<0.000001
Mono-infection vs. Polymicrobial (Poly = ref)	3.49 (1.10–11.11)	0.034	3.49 (1.10–11.11)	0.034	3.49 (1.10–11.11)	0.034
Baseline symptoms—3 vs. 2 (ref = 2)	0.083 (0.014–0.497)	0.0064	0.083 (0.014–0.497)	0.0064	0.083 (0.014–0.497)	0.0064
Baseline symptoms—4 vs. 2	—(sparse)	0.987	—(sparse)	0.987	—(sparse)	0.987

Table Note: All models are logistic mixed-effects models with a random intercept for clinical site (see Methods). Model T estimates the total (policy-relevant) effect of randomization to PCR vs. C&S (does not condition on mediators). Model M1 adds log_time to Model T (to show the effect when time is included but not appropriateness). Model M2 adds the post-randomization mediator Antibiotic Appropriateness; inclusion of this mediator substantially attenuates the adjusted arm effect while appropriateness itself is strongly associated with cure. *p*-values are two-sided Wald *p*-values. Cells marked “—” indicate variable not included or unstable due to sparse data. The intercepts differ because of covariate sets and scaling; intercepts are shown for completeness.

## Data Availability

Due to the sensitive nature of the clinical data and patient confidentiality requirements, the datasets used and/or analyzed during the current study are not publicly available. However, they are available from the corresponding author on reasonable request, provided that the request complies with relevant ethical guidelines and data protection regulations. Only Dicentra CRO had access to all data collected in this study, performed the statistical verification, and the sponsor reviewed and approved the manuscript.

## Supplementary Materials

The following supporting information can be downloaded at https://www.mdpi.com/article/10.3390/diagnostics15243107/s1.

## Abbreviations

cUTI (complicated urinary tract infection); UTI (urinary tract infection); PCR (polymerase chain reaction, here referring to rapid multiplex urinary PCR); C&S (culture and susceptibility); Mod-ITT (modified intention-to-treat); EOS (end-of-study); ITT (intention-to-treat); RCT (randomized controlled trial); NCT (ClinicalTrials.gov identifier); ACME (average causal mediation effect); ADE (average direct effect); IQR (interquartile range); OR (odds ratio); CI (confidence interval); AMR (antimicrobial resistance); LOD (limit of detection); Ct (cycle threshold).
