# Supplementary material for: Clinical Symptom Resolution Following PCR-Guided vs. Culture and Susceptibility-Guided Management of Complicated UTI: How Time-To-Antibiotic Start and Antibiotic Appropriateness Mediate the Benefit of Multiplex PCR—An Ad Hoc Analysis of NCT06996301
Source: Diagnostics (Basel). 2025 Dec 6;15(24):3107. doi: 10.3390/diagnostics15243107 (PMC12731546; doi:10.3390/diagnostics15243107)
Supplement: Supplementary file 1 [file diagnostics-15-03107-s001.zip › diagnostics-4020872-supplementary.pdf]

CLINICAL PROTOCOL COVER PAGE

**Protocol Title:**	Clinical Utility of a PCR Test for the Management of Complicated Urinary Tract Infections in Adults
**Protocol Number:**	22-UPHUV-01
**Protocol Date:**	January 12th, 2024
**Version:**	1
**Study Design:**	A multi-center, randomized, parallel-assignment, open-label, clinical utility study
**Sponsor:**	Doc Lab Inc.<br>1915 NE Stucki Ave, Suite 400<br>Hillsboro, Oregon, 97006
**CRO:**	10343781 Canada Inc. (dba dicentra CRO)<br>603-7 Saint Thomas Street<br>Toronto, Ontario, M5S 2B7<br>Canada

Protocol Signatures:

Parties	Name	Signature	Date
**Sponsor:**<br><br>Doc Lab Inc.<br>1915 NE Stucki Ave, Suite 400<br>Hillsboro, Oregon, 97006			
**Principal Investigator:**<br><br>Thomas Huard, PhD<br>Doc Lab Inc.<br>1915 NE Stucki Ave, Suite 400<br>Hillsboro, Oregon, 97006			
**Site Investigator:**			

Protocol Revisions:

Version #	Description of Changes	Date	Signature
0	Original (consult QMS for signed version)	Signed on 06/23/23	Signed by Dr. Thomas K. Huard
1	• Addition of the appendix V. Sub-study: Negative Patient Testing<br>• Addition of exploratory endpoint and analysis plan (sub-study data analysis)<br>• Addition of secondary endpoint (discordant analysis)		

Table of Contents

1. LIST OF ABBREVIATIONS 5
2. PROTOCOL SUMMARY 7
2.1. Synopsis 7
2.2. Schedule of Assessments 11
3. BACKGROUND AND RATIONALE 12
4. STUDY OBJECTIVES AND ENDPOINTS 13
5. STUDY DESIGN 15
5.1. General Design 15
5.2. Schematic of Study Design 16
5.3. Estimated Study Duration 16
5.3.1. Study Duration for Subjects 16
5.3.2. End of Study 16
5.4. Interim Analysis 17
6. SUBJECT SELECTION AND WITHDRAWAL 17
6.1. Inclusion Criteria 17
6.2. Exclusion Criteria 18
6.3. Prohibited Prior or Concomitant Therapy 18
6.4. Procedure and Consequence for Subject Withdrawal from Study 19
7. STUDY TREATMENTS 19
7.1. Subject Enrollment 19
7.2. Testing Site Setting 19
7.3. Study Arms 19
7.4. Randomization 20
7.5. Blinding and Unblinding 20
8. STUDY VISITS 20
8.1. Pre-screening 20
8.2. Visit 1 - Screening and Baseline (Day 0) 21
8.3. Visit 2 - Treatment (Day 0 + 5) 21
8.4. Visit 3 - End-of-Study (Day 28 ± 7) 22
9. ASSESSMENTS AND PROCEDURES 23
9.1. Urine Sample Analysis 23
9.2. Body Temperature 24
9.3. Blood Pressure and Heart Rate 24
9.4. Physical Examination 24
9.5. Termination of the Study 24
10. SAFETY INSTRUCTIONS AND GUIDANCE 24
10.1. Definitions of Adverse Events (AEs) 24
10.2. Collecting, Recording, and Reporting of AEs 25
10.3. Obligations of the Sponsor 27

10.4. Adverse Events Monitoring ... 27
10.5. Unscheduled Visits ... 27
11. STATISTICAL EVALUATION ... 27
11.1. Sample Size ... 27
11.2. Study Population ... 28
11.3. Analysis Plan ... 28
11.3.1. Analysis Population ... 28
11.3.2. Primary, Secondary and Exploratory Endpoints ... 29
11.3.3. Statistical Analysis of the Primary Endpoint ... 29
11.3.4. Statistical Analysis of Secondary Endpoints ... 30
11.3.5. Statistical Analysis for the Exploratory Endpoints: ... 31
12. PROTOCOL DEVIATION(S) ... 31
13. PROTOCOL CHANGES ... 31
14. ETHICAL CONSIDERATIONS ... 31
14.1. Ethical Conduct of the Study ... 31
14.2. IRB Approval ... 31
14.3. Informed Consent Form (ICF) ... 32
14.4. Risks and Procedures to Minimize Risk ... 32
15. QUALITY ASSURANCE AND QUALITY CONTROL ... 33
15.1. Auditing and Inspecting ... 33
15.2. Study Monitoring ... 33
15.2.1. Responsibilities of the Investigator(s) ... 33
15.2.2. Responsibilities of the Sponsor ... 33
15.2.3. Source Document Requirements ... 34
16. DATA HANDLING AND RECORD KEEPING ... 34
16.1. Confidentiality ... 34
16.2. Source Data and Source Documents ... 35
16.3. Case Report Forms (CRFs) ... 35
16.4. Data storage and Access ... 35
16.5. Data Quality Assurance ... 36
17. REFERENCES ... 37
18. APPENDICES ... 38
Appendix I. Treating Investigator Questionnaire ... 38
Appendix II. Urine Sample Collection ... 40
Appendix III. Device specification: pathogen targets and Cq ranges, antibiotics resistance genes and Cq ranges ... 41
Appendix IV. Comparator test results ... 42
Appendix V. Sub-study: Negative Patient Testing ... 45

1. LIST OF ABBREVIATIONS

AE	Adverse Event
CFU	Colony-Forming Unit
CKD	Chronic Kidney Disease
C_q	Quantitative Cycle
CRF	Case Report Form
CRO	Clinical Research Organization
CTMS	Clinical Trial Management Software
cUTI	Complicated Urinary Tract Infection
CVA	Costovertebral Angle
C&S	Culture and Sensitivity
DBP	Diastolic Blood Pressure
DOB	Date of Birth
Df	Degree of Freedom
eCRF	Electronic Case Report Form
EDA	Exploratory Data Analysis
EDC	Electronic Data Capture (Platform)
EOS	End of Study
ET	Early Termination
FCl	Favorable Clinical Outcome
GCP	Good Clinical Practice
H_0	Null Hypothesis
H_1	Alternative Hypothesis
HIPAA	Health Insurance Portability and Accountability Act
Hpf	High Power Field
HR	Heart Rate
IB	Investigator’s Brochure
ICF	Informed Consent Form
ICH	International Council for Harmonization
IFU	Instruction for Use
IRB	Institutional Review Board
ITT	Intention-to-Treat
Mod-ITT	Modified Intent-To-Treat Population
NCS	Not Clinically Significant
OTC	Over-the-Counter
PCR	Polymerase Chain Reaction
PD	Protocol Deviation
PI	Principal Investigator
PKD	Polycystic Kidney Disease
PV	Protocol Violation

RT-PCR	Reverse Transcriptase Polymerase Chain Reaction
SAE	Serious Adverse Event
SBP	Systolic Blood Pressure
SIV	Site Initiation Visit
SOP	Standard Operating Procedure
TAT	Turnaround Time
TEAE	Treatment Emergent Adverse Event
TLF	Tables, Listings and Figures
UTI	Urinary Tract Infection
WBC	White Blood Cell

2. PROTOCOL SUMMARY

(For Sub-study – see *Appendix V*)

2.1. Synopsis

**Title**	Clinical Utility of a PCR Test for the Management of Complicated Urinary Tract Infections in Adults
**Protocol Number**	22-UPHUV-01
**Study Center(s)**	At least 6 study sites located in the United States
**Study Design**	Multi-center, randomized, parallel-assignment, open-label, clinical utility study
**Sample Size**	N=720 (120 per site)
**Study Objectives**	**Primary Objective:**<br>• To evaluate patients' symptomatic responses following diagnosis and treatment based on the results of the molecular method versus those from the conventional urine culture method<br><br>**Secondary Objectives:**<br>• To determine the efficacy of antibiotic selection using molecular method versus the conventional urine culture method<br>• To assess clinical utility of Polymerase Chain Reaction (PCR) results during the clinical decision-making phase in patient care<br>• To compare the availability of the antimicrobial susceptibility information from molecular testing and conventional testing at the time of initial antimicrobial therapy<br>• To quantify the overall agreeability between the diagnostic results generated by PCR versus Culture and Sensitivity (C&S)<br>• To assess the symptomatic responses of patients with discordant results [PCR(+), CS(-) and PCR(-), CS(+)]<br><br>**Exploratory Objectives** (*Appendix V*) **:**<br>• To assess the effectiveness of PCR in avoiding detection of clinically insignificant infections in comparison to the performance of C&S
**Study Population**	Adult patients with suspected* Complicated Urinary Tract Infections (cUTI)

	*Symptomatic patients with evidence of pyuria AND at higher risk for Urinary Tract Infection (UTI) complications. In this study, patient population considered at higher risk are:<br>1) Elderly (≥65 years) or;<br>2) Male or;<br>3) Pregnant or;<br>4) Recurrent UTI – defined as multiple occurrence (≥3) of uncomplicated or complicated UTI in past 12 months, despite adequate treatment or;<br>5) Underlying co-morbidities: metabolic disorder (e.g. diabetes), immunosuppression, or impaired renal function (e.g. chronic kidney disease (CKD)) or;<br>6) Known functional and anatomic abnormalities of the urinary tract (e.g. stones, stents, recent instrumentation, indwelling catheters, neurogenic bladder, or polycystic kidney disease (PKD)) or;<br>7) Persistent infection after primary antibiotic therapy
**Inclusion Criteria**	*Subjects must meet all of the following criteria to be considered eligible for admission to the study:*<br>I1. At least 18 years of age at the time of consent;<br>I2. Presenting at least two of the following new, persistent or worsening cUTI signs and symptoms at screening visit:<br>a) fever (temperature >38 degrees Celsius or >100.4 degrees Fahrenheit), hypothermia (temperature <35.5 degrees Celsius or <95.9 degrees Fahrenheit), rigors, or chills<br>b) dysuria, urinary frequency, urgency, or hematuria<br>c) suprapubic pain or pelvic pain<br>d) costovertebral angle (CVA) tenderness<br>e) nausea or vomiting<br>f) radiographic evidence of pyelonephritis<br>g) leukocytosis<br>I3. Urine specimen with evidence of pyuria<br>a) dipstick analysis positive for nitrite and/or leukocyte esterase, or;<br>b) ≥10 white blood cells (WBCs) per cubic millimeter [mm^3], or;<br>c) ≥10 WBCs per high power field (hpf), or;<br>d) clinically suspected pyuria (e.g. change in urine color, sediment in urine, or foul-smelling urine)

	I4. Having cUTI that requires microbiological diagnosis and treatment as suspected by the Investigator;<br>I5. Presenting active UTI that failed to resolve on first-line therapy or identified as a high-risk* patient population;<br>*High-risk patient population include those who are elderly (≥65years), male, pregnant, having recurrent UTI (≥3/year), with underlying co-morbidities (e.g. diabetes, immunosuppression, or CKD), or with known functional and anatomical abnormalities of the urinary tract (e.g. stones, stents, recent instrumentation, indwelling catheters, neurogenic bladder, or PKD)<br>I6. Able to provide at least 8 mL urine at visit 1 and 3;<br>I7. Willing to abstain from sexual intercourse or use condoms during any sexual contact until the End-of-Study (EOS) visit is complete;<br>I8. Willing to comply with protocol requirements, including availability for follow-up for the duration of the study.
**Exclusion Criteria**	*Subjects meeting any of the following criteria will not be eligible for admission to the study:*<br>E1. Unable or unwilling to provide written informed consent;<br>E2. Unable to read and write in English (surveys are not available or validated in any other language than English);<br>E3. Currently participating in or has participated in an interventional clinical trial with an investigational product or device within 30 days prior to the Screening Visit;<br>E4. Currently on or chronic use of any antibiotics for any clinical indication, other than UTI (refer to section 6.3.);<br>E5. Receipt of any dose of a potentially therapeutic oral or systemic antibiotics for the treatment of UTI within 48 hours before the study baseline urine is obtained<br>E6. Pregnant women with known fetal congenital anomaly (e.g., genetic abnormality or major congenital malformation) based on antenatal ultrasound;<br>E7. Any rapidly progressing disease or immediately life-threatening illness, including acute hepatic failure, or respiratory failure<br>E8. Medical condition or other factor that in the judgment of the investigator might affect ability to comply with procedures.
**Study Duration**	The duration of study participation for each enrolled patient is approximately 28 days.

**Study Endpoints**	**<u>Primary Endpoint:</u>**<br>• Number (and percentage) of patients in each study arm with favorable clinical outcomes* at the EOS visit<br>*favorable clinical outcomes are defined as a clinical response of improvement[a] and/or cure[b]<br>[a] Clinical improvement is defined as 1) Resolution of cUTI signs and symptoms present at baseline, 2) Development of no new cUTI symptoms and/or 3) Avoidance of parenteral antibiotic therapy, in or out of hospital, at any time after randomization<br>[b] Clinical cure is resolution of all acute signs and symptoms of cUTI and improvement to such an extent that no further antimicrobial therapy (IV or oral) is required for the treatment of the cUTI<br><br>**<u>Secondary Endpoints:</u>**<br>• Number (and percentage) of patients in each arm with microbiological eradication** of all baseline pathogens at the EOS visit<br>**microbiological eradication of all baseline pathogens is defined as an end of study quantitative urine culture that shows all uropathogens found at baseline are reduced to $<10^5$ CFU/mL and all baseline pathogens are not detected by EOS urine PCR (Cq>33)<br>• Subjective measurement of Treating Investigator Satisfaction Score as evaluated by the questionnaire at EOS<br>• Turnaround Time (as measured in hours) of molecular diagnostic procedures compared to conventional diagnostics (time from collection of samples to complete identification of organism(s) and availability of antimicrobial susceptibility results)<br>• Overall agreeability between the diagnostic results generated by PCR versus C&S as assessed by discordant analysis\<br>• Assess the favorable clinical outcomes of patients with discordant results [PCR(+), CS(-) and PCR(-), CS(+)]<br><br>**<u>Safety:</u>**<br>Assessments of safety will include the following:<br>• Clinical observations<br>• Vital sign measurements<br>• Laboratory tests<br>• Reported adverse events

	• Assessment of Treatment Emergent Adverse Events (TEAE) at EOS<br><br>**Exploratory Endpoints** (*Appendix V*)**:**<br>• The negative percent agreement of the PCR test (specificity) compared to C&S in not detecting clinically insignificant UTIs
**Study Arms**	*Active Comparator A:* Diagnosis by Molecular Testing - Treatment based on the results of Urine PCR testing – even numbers on randomization table.<br>• KingFisher Duo Prime<br>• QuantStudio 6 and 7 Flex Real-Time PCR System<br>• QuantStudio 12k Flex Real-Time PCR System<br><br>*Active Comparator B:* Diagnosis by Conventional Testing - Treatment based on the results of Urine C & S testing – odd numbers on randomization table
**Statistical Methodology**	Tables, listings and figures (TLFs) will be utilized to summarize data associated with endpoints; discussion sections will be based on observations of this summarized data, with reference to existing literature where appropriate.

2.2. Schedule of Assessments

The schedule of assessments table provides an overview of the protocol visits and procedures. The investigator may schedule visits (unscheduled visits) in addition to those listed on the schedule of assessments, in order to conduct evaluations or assessments required to protect the well-being of the subject.

Table 1. Schedule of Assessments

Assessments	Screening and Baseline V1 (Day 0)	Treatment[i] V2 (Day 0 to 5)	End-of-Study V3 (Day 28 ± 7)	Unscheduled Visit[i] UV
Informed Consent	x			
Eligibility Criteria	x	x		
Demographics[a]	x			
Medical history	x			
Vital signs[b]	x		x	x
Physical Examination	x		x	x
Concomitant therapy	x	x	x	x
Randomization	x			

Urine sample collection	x		x	x
Urine PCR	x		x	x[e]
Urine C&S	x		x	x[e]
cUTI treatment		x[f]	x[d]	x
Adverse events[c]	x	x	x	x
Study conclusion			x	x

[i] Treatment visit (Visit 2) and/or unscheduled visits may be conducted virtually

[a] Demographics include sex, age, date of birth

[b] Vital signs include body temperature (^{0}C), SBP (mmHg), DBP (mmHg), HR (bpm) and may be conducted by the Investigator at their discretion

[c] AEs will be collected from the time of informed consent to EOS. Any ongoing AEs at EOS will be followed until resolution or stabilization

[d] cUTI treatment may continue at EOS and after patient's study exit

[e] Additional urine testing (PCR, C&S, or both) may be ordered at unscheduled visits at treating Investigator's discretion

[f] cUTI treatment must initiate within 24 hours of receiving the test results (either PCR or C&S as per subject's assigned arm)

3. BACKGROUND AND RATIONALE

Urinary Tract Infections (UTIs) are among the most common reasons for a patient to visit a clinic, and are the second most common type of bacterial infection in adults.[1] Up to 33% of all women experience a UTI in their lifetime. The majority of UTIs are caused by ascension of uropathogens from the urethra to the bladder. The most common pathogen causing UTI is *Escherichia coli*, followed by *Staphylococcus saprophyticus, Enterococcus, Klebsiella, Proteus spp, pseudomonas, Enterobacter*, and *yeast*.[2] However, a broader range of microorganisms may be responsible for UTIs and the possibility of polymicrobial infections (co-infection or super-infection) frequently complicate the clinical pictures of UTI.

The most common UTI, in a setting of functionally and structurally normal urinary tract, is uncomplicated acute cystitis. UTI is classified according to the anatomical site of infection, and distinguishes between lower tract infections of bladder (cystitis), urethra (urethritis) and upper tract infections of ureter, collecting system, and renal parenchyma (pyelonephritis).[3] Patients with risk factors are most likely to experience complicated and/or recurrent UTI that are often refractory to standard therapy and difficult to manage. Complicated UTIs (cUTIs) are frequently accompanied by local or systemic signs and symptoms, including fever, chills, rigor, malaise, back pain, flank pain, and/or costovertebral angle (CVA) tenderness, in addition to common urinary symptoms such as urgency, frequency, dysuria and/or hematuria. Conditions that increase the risk of cUTI include structural and/or functional abnormality of urinary tract,

immunosuppression, underlying metabolic disease, pregnancy, indwelling urinary catheter, azotemia caused by renal disease, etc.

UTI treatment and management affects healthcare costs and consumes vast resources in both ambulatory and inpatient settings. Outpatient management for UTIs is becoming limited due to the rising number of antimicrobial resistant cases.[4-6] As a result of predisposing risks that alter normal urinary tract (functionally or anatomically), cUTI is associated with increased risk of recurrent infection. Furthermore, the management of cUTI starts with the use of empiric broad-spectrum antimicrobial therapy that sufficiently covers the most commonly isolated uropathogens. In clinical practice, the choice of antibiotics is determined based on urine culture and antimicrobial sensitivity results. However, urine culture and sensitivity is a time-consuming procedure, where the turnaround time of the results is at least 48 hours, and this delay in a definitive diagnosis of cUTI and identification of infecting microorganisms often results in longer use of empiric broad-spectrum antimicrobial agents. An increased antimicrobial resistance in uropathogens underlying cUTI is attributed to an increased risk of recurrent infection, relapse, and exposure to multiple courses of antimicrobials, which can further complicate treatment course, prolong hospital stays, and lead to high mortality rates.[7] The use of molecular testing for the diagnosis of UTI/cUTI, such as polymerase chain reaction (PCR), can facilitate the detection of urinary pathogens with increased sensitivity and rapid turnaround times – that is typically less than 24 hours – and will enable the targeted administration of antimicrobial agents.

The proposed comparator PCR test has completed performance and clinical validation tests where it identified 15 bacterial genus and species, 4 fungal species, 5 STI-causing microorganisms, and 16 different classes of resistance genes. The study reported that all positive samples were identified via PCR at a rate of >90%, indicating that PCR may be more accurate, specific, and sensitive than culture. The clinical utilization of PCR-based diagnosis of UTI/cUTI would be extremely beneficial in ensuring timely treatment by rapid identification of infecting microorganisms and resistance, and eliminating empirical treatment by enabling early targeted treatment, ultimately reducing UTI-related morbidities and costs in a high-risk patient population.

The purpose of this study is to assess clinical utility of molecular testing in the detection of pathogens in cUTIs, identification of antimicrobial susceptibility information, and implementation of more efficacious management of cUTIs.

4. STUDY OBJECTIVES AND ENDPOINTS

This investigation intends to verify the clinical utility of urine PCR testing by comparing the diagnostic and therapeutic values of molecular diagnostic methods to traditional urine C&S in management of cUTI in adults.

A sub-study (see *Appendix V*) is planned to validate the clinical accuracy of PCR testing by comparing its effectiveness with C&S in avoiding the detection of clinically insignificant infections that may be present in healthy patients (see *exploratory endpoint*).

Table 2. Study Objectives and Endpoints

OBJECTIVES	ENDPOINTS
**Primary**	
• To evaluate patients' symptomatic responses following diagnosis and treatment based on the results of the molecular methods versus the those of conventional urine culture method	• Number (and percentage) of patients in each study arm with favorable clinical outcome* at the EOS visit<br>*favorable clinical outcomes are defined as a clinical response of improvement[a] and/or cure[b]<br>[a] Clinical improvement is defined as 1) Resolution of cUTI signs and symptoms present at baseline, 2) Development of no new cUTI symptoms and/or 3) Avoidance of parenteral antibiotic therapy, in or out of hospital, at any time after randomization<br>[b] Clinical cure is resolution of all acute signs and symptoms of cUTI and improvement to such an extent that no further antimicrobial therapy (IV or oral) is required for the treatment of the cUTI
**Secondary**	
• To determine the efficacy of antibiotic selection using molecular methods versus the conventional urine culture method<br>• To assess clinical utility of PCR results during the clinical-decision-making phase in patient care<br>• To compare the availability of the antimicrobial susceptibility information from molecular testing and conventional testing at the time of initial antimicrobial therapy<br>• To quantify the overall agreeability between the	• Number (and percentage) of patients in each arm with microbiological eradication** of all baseline pathogens at the EOS visit<br>**microbiological eradication of all baseline pathogens is defined as an end of study quantitative urine culture that shows all uropathogens found at baseline are reduced to $<10^5$ CFU/mL and all baseline pathogens are not detected by EOS urine PCR (Cq>33)<br>• Subjective measurement of Treating Investigator Satisfaction Score as evaluated by the questionnaire at EOS<br>• Turnaround Time (as measured in hours) of molecular diagnostic procedures compared to conventional diagnostics (time from collection of samples to complete identification of organism(s) and availability of antimicrobial susceptibility results)

diagnostic results generated by PCR versus C&S<br>• To assess the symptomatic responses of patients with discordant results [PCR(+), CS(-) and PCR(-), CS(+)]	• Overall agreeability between the diagnostic results generated by PCR versus C&S as assessed by discordant analysis<br>• To assess the favorable clinical outcome of patients with discordant results [PCR(+), CS(-) and PCR(-), CS(+)]
**Exploratory** (see sub-study in the *Appendix V*)	
• To assess the effectiveness of PCR in avoiding detection of clinically insignificant infections in comparison to the performance of C&S	• The negative percent agreement of the PCR test (specificity) compared to C&S in not detecting clinically insignificant UTIs

Safety will be assessed based on clinical observations, vital sign measurements, and relevant laboratory tests at applicable study timepoints. TEAE will be assessed at EOS. Participant reported adverse events will be collected throughout the study duration.

5. STUDY DESIGN

(For Sub-study – see *Appendix V*)

5.1. General Design

This is a multi-center, randomized, parallel-assignment, open-label, clinical utility study to evaluate urine PCR cUTI testing by comparing the diagnostic and therapeutic outcomes of molecular diagnostic methods to those of traditional urine C&S in management of cUTI in adults. At least six (6) sites will participate in this study. Approximately 120 eligible volunteers will be enrolled per site. The study is designed to be a 28-day study, which includes sample collection, sample test, treatment, and follow-up. Patients who meet all of the inclusion criteria and none of the exclusion criteria and sign an informed consent form will be enrolled in the study. During the visit, each enrolled patient will provide a urine sample (at least 8 mL) at V1 and V3; The collected urine specimens will be analyzed using both PCR and C&S methods at each timepoint. Upon eligibility confirmation by treating Investigator(s), patients will be randomized in a 1:1 ratio as follows:

- Diagnosis and treatment based on the results of the urine PCR
- Diagnosis and treatment based on the results of the urine C&S

Table 1. Schedule of Assessments summarizes the study procedures to be performed at each study visit. Individual study procedures are described in detail below (see section 8). It may be

required to perform outlined procedures at unscheduled time points, if deemed clinically necessary by the Investigator(s). Additional evaluation/testing may be deemed necessary by the Investigator(s) for reasons related to subject safety.

5.2. Schematic of Study Design

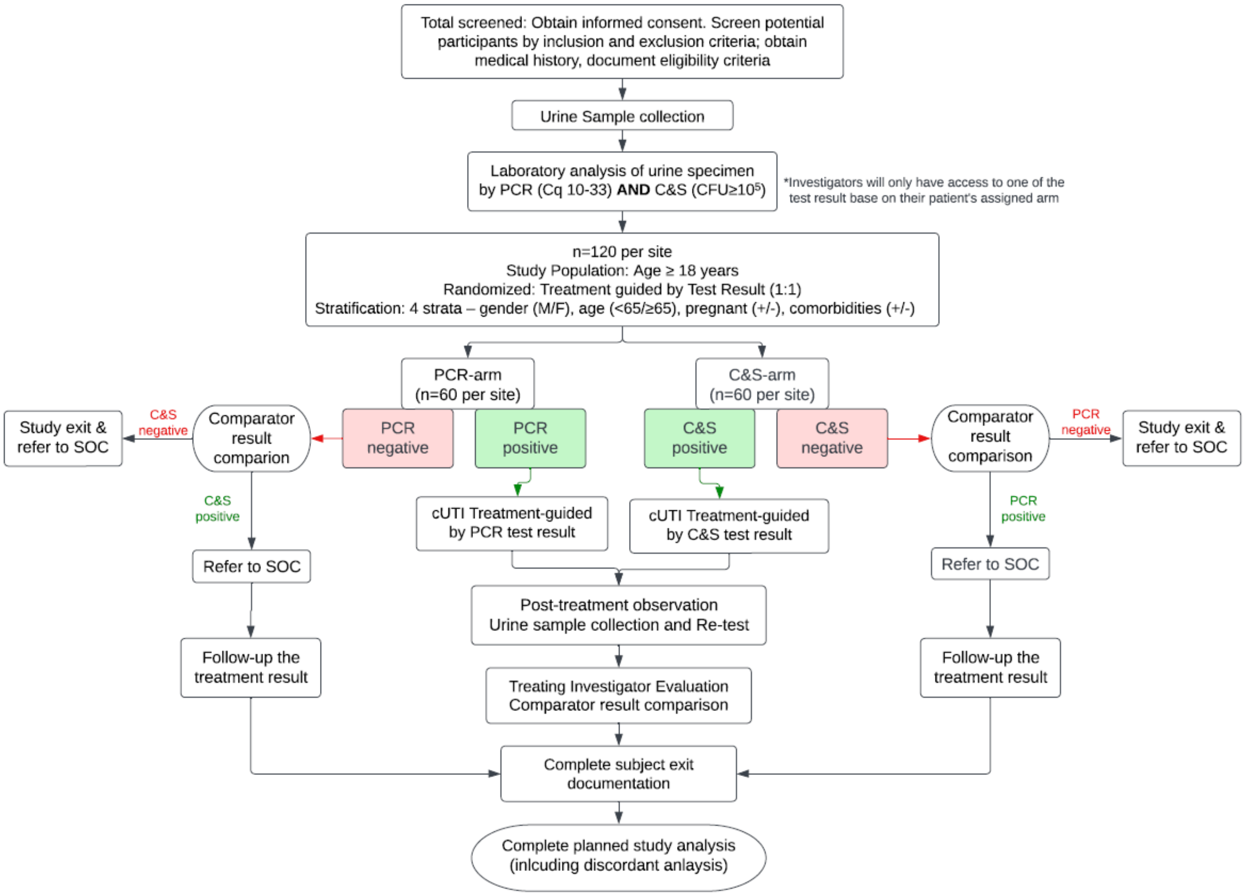

Note: SOC treatment will be determined based on the diagnostic evidences and the treating Investigator's judgement

5.3. Estimated Study Duration

5.3.1. Study Duration for Subjects

The duration of study participation for each enrolled subject is approximately 28 days.

5.3.2. End of Study

The end of study is defined as when the subject completes the last study-related visit/contact, withdrawal from the study, or is lost to follow-up. The last subject enrollment will occur when an adequate number of subjects that satisfy the study endpoint criteria are achieved.

5.4. Interim Analysis

No interim analysis is planned in this study.

6. SUBJECT SELECTION AND WITHDRAWAL

(For Sub-study – see *Appendix V*)

6.1. Inclusion Criteria

Subjects must meet all of the following criteria to be considered eligible for admission to the study:

- I1. At least 18 years of age at the time of consent;
- I2. Presenting at least two of the following new, persistent or worsening cUTI signs and symptoms at screening visit:
  - a) fever (temperature >38 degrees Celsius or >100.4 degrees Fahrenheit), hypothermia (temperature <35.5 degrees Celsius or <95.9 degrees Fahrenheit), rigors, or chills
  - b) dysuria, urinary frequency, urgency, or hematuria
  - c) suprapubic pain or pelvic pain
  - d) costovertebral angle (CVA) tenderness
  - e) nausea or vomiting
  - f) radiographic evidence of pyelonephritis
  - g) leukocytosis
- I3. Urine specimen with evidence of pyuria
  - a) dipstick analysis positive for nitrite and/or leukocyte esterase, or;
  - b) ≥10 white blood cells (WBCs) per cubic millimeter [mm3], or;
  - c) ≥10 WBCs per high power field (hpf), or;
  - d) clinically suspected pyuria (e.g. change in urine color, sediment in urine, or foul-smelling urine)
- I4. Having cUTI that requires microbiological diagnosis and treatment as suspected by the Investigator;
- I5. Presenting active UTI that failed to resolve on first-line therapy or identified as a high-risk* patient population;

  *High-risk patient population include those who are elderly (≥65years), male, pregnant, having recurrent UTI (≥3/year), with underlying co-morbidities (e.g. diabetes, immunosuppression, or CKD), or with known functional and anatomical abnormalities of

the urinary tract (e.g. stones, stents, recent instrumentation, indwelling catheters, neurogenic bladder, or PKD)

I6. Able to provide at least 8 mL urine at visit 1 and 3;

I7. Willing to abstain from sexual intercourse or use condoms during any sexual contact until the EOS visit is complete;

I8. Willing to comply with protocol requirements, including availability for follow-up for the duration of the study.

6.2. Exclusion Criteria

Subjects meeting any of the following criteria will not be eligible for admission to the study:

E1. Unable or unwilling to provide written informed consent;

E2. Unable to read and write in English (surveys are not available or validated in any other language than English);

E3. Currently participating in, or has participated in an interventional clinical trial with an investigational product or device within 30 days prior to the Screening Visit;

E4. Currently on or chronic use of any antibiotics for any clinical indication, other than UTI (refer to section 6.3.);

E5. Receipt of any dose of a potentially therapeutic oral or systemic antibiotics for the treatment of UTI within 48 hours before the study baseline urine is obtained

E6. Pregnant women with known fetal congenital anomaly (e.g., genetic abnormality or major congenital malformation) based on antenatal ultrasound;

E7. Any rapidly progressing disease or immediately life-threatening illness, including acute hepatic failure, or respiratory failure;

E8. Medical condition or other factor that in the judgment of the investigator might affect ability to comply with procedures.

6.3. Prohibited Prior or Concomitant Therapy

At screening, the Investigator or delegate will review prior medications use and record all prior medication taken by the subject within 7 days before the screening (or within 14 days before the screening for all antimicrobial agents, and within 30 days before the screening for any other investigational treatment).

Patients with current or chronic use (as defined as medication taken daily for ≥30 days or used on an “as needed” basis for ≥6 months) of any antibiotics for any clinical indications, other than UTI, will be excluded from the study. Patients will be excluded from the study if they have received any dose of a potentially therapeutic oral or systemic antibiotics for the treatment of UTI within 48 hours before the study baseline urine is obtained.

6.4. Procedure and Consequence for Subject Withdrawal from Study

<u>Removal by Investigator:</u>
The removal of a study patient by the Investigator will be based on the Clinical and Protocol Violation conditions outlined below. Criteria for patient removal at the Investigator's discretion are:

<u>Clinical</u>
A patient may be withdrawn from the study if, in the opinion of the Investigator, it is not in the best interest of the patient to continue. Patients who experience an adverse event (AE) or severe adverse event (SAE) will be assessed by the Investigator to determine disposition.

<u>Protocol Violation</u>
Protocol violations will be assessed by the Investigator on a case-by-case basis to determine subject disposition.

Patients can be withdrawn from the study by the Investigator at any time, or upon request from the Sponsor and with mutual agreement between sponsor and Investigator. The Investigator and/or delegate must inform the patient and/or Sponsor of the removal and provide a rationale.

7. STUDY TREATMENTS

(For Sub-study – see *Appendix V*)

7.1. Subject Enrollment

Each site will recruit and screen approximately 168 adults to identify the defined study population (n=120) based on an assumed 40% screen failure rate.
Patients will have their eligibility assessed by the Investigator and/or delegate, through the review of relevant medical history and other assessments as defined in the protocol.

7.2. Testing Site Setting

The testing sites shall be high-complexity testing laboratories within clinical practices.

7.3. Study Arms

<u>Active Comparator A</u>
Diagnosis by Molecular Testing, and Treatment based on the results of Urine PCR testing – EVEN numbers on randomization table.

Active Comparator B
Diagnosis by Conventional Testing, and Treatment based on results of the Urine C & S testing – ODD numbers on randomization table.

7.4. Randomization

Enrolled patients will be randomized for one of two testing procedures used to guide treatment. Upon eligibility confirmation, patients will be assigned a unique sequential enrollment/randomization number. Patients with an odd enrollment number will receive treatment guided by urine C&S results, and patients with an even enrollment number will receive treatment guided by urine PCR results.

Enrollment numbers will only be used once. If a patient withdraws from participation in the study, then their enrollment number cannot be reused. Enrollment numbers will be assigned strictly sequentially as potential patients become eligible for randomization.

7.5. Blinding and Unblinding

The treating Investigator will only have access to the results of their patients' assigned test, and will be blinded to the result of the comparator test until after the end of the study.

The treating Investigator shall gain an access to the result of comparator test in following cases:

- If a patient's assigned test result comes back negative UTI.
- If a patient is withdrawn from the study. In the opinion of the Investigator, it is not in the best interest of the patient to continue study therapy and/or lack of efficacy.
- If patient decide to discontinue study participation.

8. STUDY VISITS

(For Sub-study – see *Appendix V*)

8.1. Pre-screening

Recruitment will commence once the clinical site has received approval from the IRB, and staff have been delegated to perform recruitment activities. Patients may self-present by contacting the study team as per the IRB-approved recruitment materials to complete the pre-screening procedure, at which point they will be scheduled for a screening and baseline visit. Any recruitment materials used will be approved by applicable regulatory bodies prior to distribution and/or use.

8.2. Visit 1 - Screening and Baseline (Day 0)

The delegated study staff will explain the study objectives, the process, and the modalities of participation in the research. The study staff will answer any questions the patient may have. Patients will then be given ample time to review the Informed Consent Form (ICF) and, ask any questions before signing the appropriate section(s). The ICF will then be signed by the Investigator or delegate. A copy of the Informed Consent Form (ICF) will be provided to the patient for their records. The informed consent process will be documented in the source documents.

Once the ICF is signed by the patient and attested to by the Investigators or delegate, the study staff will collect demographic information, vital signs and relevant medical history, and conduct an examination in order to verify the eligibility criteria (excluding urine analysis results).

If the patients meet the inclusion criteria and none of the exclusion criteria (excluding urine analysis results), urine specimens will be collected using acceptable methods (refer to section 9.1). The collected urine specimens will be analyzed by both urine PCR and C&S in accordance with SOPs.

Prior to Visit 2 and upon confirmation of eligibility by an Investigator, patients will be enrolled into the study and will be randomized into one of two study arms (PCR-arm or C&S-arm).

8.3. Visit 2 - Treatment (Day 0 + 5)

Visit 2 **MUST** initiate within 24 hours of the test result (urine C&S or urine PCR as applicable to subject's assigned arm) becoming available and within specified buffer window. Study staff will record the data associated with the PCR test report or C&S test report in a dedicated eCRF, including time of the test reports.

Visit 2 may be conducted virtually, as per treating Investigator's discretion, if the patient care can be continued in an out-patient clinic/setting.

The identity of the patient will be confirmed by verbal verification of DOB and name. The Investigator or delegate will review medical history. Patients will be asked about any changes in their health, and any concomitant treatments administered since the last visit.

The treating Investigator will utilize the results of the patients' randomly assigned study arm for their clinical decision making and patient care. The treating Investigator **MUST NOT*** review the results of the (unassigned) comparator test before treatment.

**Exception: If the patient's assigned test detects no UTI, the treating Investigator will review the result of the comparator test. In the case of concordant result between two comparators (both negative), the patient will be referred to SOC and excluded from the study. In the case of discordant result, the patient will be referred to SOC and the result of patient care will be followed up by the Investigator or delegate. The patient data will be collected and used for discordant analysis as planned (refer to section 11.3.4).*

The treating Investigator shall provide continuous patient care as per their medical decision(s). These include, but are not limited to: request of unscheduled visits, admission/referral of patient to receive in-patient care, administration of IV or oral antibiotics, in/out-patient care, or use of broad-spectrum antibiotics.

8.4. Visit 3 - End-of-Study (Day 28 ± 7)

EOS assessment should be performed in-person within the specified buffer period.
The Investigator or delegate will perform/collect the following:

- Vital signs
- Physical examination (may be complete, targeted, or omitted; discretion of the Investigator)
- Record(s) of any adjunctive therapeutic procedures performed
- Record(s) of concomitant medications
- Assessment of clinical outcome(s)
- Record(s) of any AEs/SAEs

Urine specimens will be collected for microbiological evaluation. In case the result of urine specimen identifies an unsuccessful treatment, the patient may be followed-up to verify the final clinical outcome if deemed necessary by the treating Investigator.

When both the results of urine C&S and urine PCR become available, the treating Investigator will answer an evaluation questionnaire concerning his/her judgement of the diagnostic test and therapeutic value of urine PCR and urine C&S.

In case of clinical failure* as determined by treating Investigator at EOS, the patient's condition will be considered an AE and followed-up accordingly.
*Clinical failure is defined as a patient who has 1) on-going AE/SAE at EOS and/or 2) persistent cUTI symptoms and/or 3) developed new symptoms of cUTI.

At the end of the study, the Investigator will complete the Study Exit Form present at the end of the eCRF portfolio. This form must also be completed in the event of premature termination of

the patient's participation should they, the Investigator, or the Sponsor choose to discontinue their participation.

9. ASSESSMENTS AND PROCEDURES

9.1. Urine Sample Analysis

Urine samples (at least 8 mL) will be collected from a clean-catch mid-stream urine specimen at V1and V3. Patients will be instructed to follow the clean catch instructions provided at the collection site. The Investigator or delegate may collect the urine samples using a properly disinfected collection techniques such as through a newly-placed urinary catheter, cystoscopy, or suprapubic aspiration, as per clinical indication (See Appendix II for further procedural guidance). Additional urine sample collection and analysis may be conducted at an unscheduled visit, if deemed necessary by treating Investigator. Specimens must be immediately refrigerated upon collection and submitted to the central laboratory within 24 hours of collection. Specimen must be stored and maintained at 2-8 ºC when transporting. If transportation to the central laboratory is expected to exceed 24 hours, whole cups of urine specimen must be frozen.

Obtained urine samples will be labelled with subject ID, DOB, visit number, study ID, method of collection, date and time of collection. The collected sample will be placed in a small bag along with the test requisition form. The processing laboratory will aliquot the sample upon receipt, one to be analyzed by PCR (maximum 3mL) and the other to be analyzed by C&S (minimum 5mL). The sample will be stored under the appropriate temperature conditions until analysis of the sample.

Urine C&S

All urine samples for Urine C&S will be shipped to the central laboratory. Urine culture, isolation of uropathogen(s), initial identification of pathogen(s) and bacterial counts in urine will be conducted in the central laboratory.

Urine samples will be cultured and quantified using a calibrated loop to identify a quantitative count of bacteria at a lower limit of 10^5 CFU/mL. All purified pathogen(s) will be further analyzed for species identification and antimicrobial susceptibility. The analysis results will be recorded in the source document and eCRF.

Urine PCR

Urine samples will be analyzed using the listed molecular testing devices:

- KingFisher Duo Prime
- QuantStudio 6 and 7 Flex Real-Time PCR System
- QuantStudio 12k Flex Real-Time PCR System

Bacterial and fungal pathogen-directed qualitative PCR amplification of target region of

24 uropathogens, and 16 different classes of resistance genes (see Appendix III. Device specification: pathogen targets and Cq ranges, antibiotics resistance genes and Cq ranges).

Further details of the standardized SOP for PCR procedure will be distributed from a central laboratory to all laboratory sites prior to the commencement of the study.

9.2. Body Temperature

Body temperature will be measured at Visit 1, Visit 3, and possibly at unscheduled visits. Temperature should be measured orally, rectally, or by tympanic route. Temperature should be assessed as per institution guidelines while at the study site, and recorded in the eCRF.

9.3. Blood Pressure and Heart Rate

Blood Pressure and Heart Rate will be measured at Visit 1, Visit 3, and possibly at unscheduled visits. Blood Pressure and Heart Rate will be measured according to the site's SOPs. Additional Blood Pressure and Heart Rate collections or changes to collection times will be permitted, as necessary, at the discretion of the Investigator to ensure proper collection of safety data. Appropriately sized and calibrated equipment will be used at each measurement of Blood Pressure and Heart Rate.

9.4. Physical Examination

The Investigator or delegate will perform a physical examination at V1 and V3. The physical examination may be complete, targeted, or omitted at the Investigator's discretion, based on the patient's condition and circumstances.

9.5. Termination of the Study

The study may be terminated at any time by the Sponsor, PI, or applicable regulatory authority (IRB). If the study is terminated prematurely, the PI/Investigator(s), patients, and the regulatory authorities must all be notified of the termination promptly. Upon termination of the study whether premature, or due to completion, site close-out activities will be initiated including regulatory close-out to the appropriate authorities.

10. SAFETY INSTRUCTIONS AND GUIDANCE

10.1. Definitions of Adverse Events (AEs)

10.1.1. Adverse Event

Definition: An adverse event can be any unfavorable and unintended sign, symptom, or disease that happens during the study participation (defined as after the time of initial informed consent), whether or not it is considered study drug, device, or procedure-related. Adverse event (AE) may be mild, moderate, or severe, and may be caused by something other than the drug or therapy being given. A pre-existing condition is one that is present at study entry and is reported as part of the subject's medical history; if the frequency, intensity, or character of the condition worsens during the study participation, it should be reported as an AE. Any abnormality that presents during a medical test is to be defined as an AE if it produces clinical signs and/or symptoms, requires intervention, or deemed clinically significant by the investigator. Subject should be instructed to report all AEs to the investigators or study staff. Adverse events must be followed-up to resolution or when the condition is deemed stable by the Investigator.

10.1.2. Serious Adverse Event

A Serious Adverse Event (SAE) is any AE, regardless of causality that results in any of the following:

- Death;
- Life-threatening AE;
- Requires inpatient hospitalization;
- Persistent or significant disability/incapacity or;
- Medical event that may jeopardize the patient/subject and may require medical or surgical intervention.

10.2. Collecting, Recording, and Reporting of AEs

10.2.1. Collecting and Recording

The Investigator or delegated study staff must record all adverse events in an AE form with information about:

- Details of adverse event
- Date of onset (time can be recorded, if applicable)
- Intensity (mild, moderate, severe)
- Causal relationship to study involvement (probable, possible, unlikely, not related)
- Other actions taken
- Date and time of outcome
- Outcome

All AEs, regardless of causal relationship to study involvement, spanning from the signature of the informed consent form until the end of the study as defined by the protocol for that subject, are to be recorded on the corresponding eCRF.

10.2.2. Guidelines for Reporting Adverse Events

The following timelines apply to the reporting of AEs/SAEs as applicable. The Investigator must notify the Sponsor:

- Within 24 hours of the Investigator becoming aware of the event if it results in death of a patient
- Within 2 weeks (10 business days) after becoming aware of the event if:
  - It is an AE which is related to the conduct of the study;
  - It is an AE that is expected (listed in the ICF as a potential side effect) but is occurring more frequently than expected;
  - It is an unexpected AE/SAE that is related to the conduct of the study but is not life-threatening.
- Annually or upon study completion (together with the Study Status Report) if:
  - It is an expected AE (listed in the ICF as a potential side effect);
  - It is an unexpected AE that is unlikely to be related to the conduct of the study and is not life-threatening.

During the course of a clinical study, the sponsor shall notify the IRB of any suspected adverse reaction to study treatment that is both serious and unexpected:

- A single occurrence of a serious, unexpected event that is uncommon and strongly associated with drug exposure;
- A single occurrence, or more often a small number of occurrences, of a serious, unexpected event that is not commonly associated with drug exposure, but uncommon in the study population;
- Multiple occurrences of an AE that, based on an aggregate analysis, is determined to be an unanticipated problem;
- An AE that is described or addressed in the investigator's brochure, protocol, or informed consent documents, but occurs at a specificity or severity that is inconsistent with prior observations;
- A serious AE that is described or addressed in the investigator's brochure, protocol, or informed consent documents, but for which the rate of occurrence in the study represents a clinically significant increase in the expected rate of occurrence;
- Any other AE or safety finding that would cause the sponsor to modify the investigator's brochure, study protocol, or informed consent documents, or would prompt other action by the IRB to ensure the protection of human subjects.

10.3. Obligations of the Sponsor

During the course of study, the Sponsor will report all SAEs that are both unexpected and considered related to Investigational device to the regulatory authorities and/or IRBs as appropriate and to the Investigators. The Sponsor will report all SAEs that are expected and considered related to the investigational device to the regulatory authorities, according to the local regulations. The Sponsor will report all safety observations made during the conduct of the study in the clinical study report.

10.4. Adverse Events Monitoring

All events will be managed promptly and reported in compliance with all applicable regulations and guidelines, and will be included in the final clinical study report.

10.5. Unscheduled Visits

Patients may contact delegated study staff about possible changes in their health that are more than minor and/or that persist; or delegated study staff may contact patients when additional assessments are deemed needed. Patients may be asked to return to the clinic to repeat laboratory tests or for additional assessment if requested by the Investigator. In these cases, such evaluations/testing will be performed in accordance with those applicable regulations.

11. STATISTICAL EVALUATION

(For Sub-study – see *Appendix V*)

11.1. Sample Size

The sample size calculation was conducted using the software package G*Power (v3.1.9.7) on a two-group independent proportions Fisher's exact-test (one-tailed), chosen as the statistical test for the primary endpoint of this study. A type I (α) error probability of 0.05, and a power ($1 - \beta$) of probability 0.85 was chosen for the calculation; an allocation ratio of 1:1 was set to address the equally-sized treatment arms.

According to literature, patient populations that used C&S testing for cUTI management has a favorable clinical outcomes (FCl) percentage ranging from 60%-90% [8]. For the purpose of this study, we assumed an FCl percentage of 75% for the C&S arm. A 10% expected improvement of the FCl in the PCR arm will require 574 enrolled participants. Assuming a 25% attrition rate & post-hoc exclusion rate (due to PCR or C&S negative result after randomization), up to 146 additional participants will be enrolled. Thus, up to 720 participants will be sufficient for

completion of the study. The participants will be enrolled in at least 6 study sites (with an equal distribution of participants enrolled per site).

Exact – Proportions: Inequality, two independent groups (Fisher's exact test)

**Options**:	Exact distribution		
**Analysis**:	A priori: Compute required sample size		
**Input**:	Tail(s)	=	One
	Proportion p1	=	0.7
	Proportion p2	=	0.8
	α err prob	=	0.05
	Power (1–β err prob)	=	0.85
	Allocation ratio N2/N1	=	1
**Output**:	Sample size group 1	=	287
	Sample size group 2	=	287
	Total sample size	=	574
	Actual power	=	0.8513193
	Actual α	=	0.0404397

11.2. Study Population

The Intention-to-Treat (ITT) population: All participants who participate in the study and get randomized into one of the study's arms, without any major protocol violations which would significantly compromise the integrity of collected data.

The Modified Intent-To-Treat (Mod-ITT) Population: All participants from the ITT with a positive PCR or C&S results after randomization assignment.

The Exploratory Population: All participants who participate in the Negative Patient Testing sub study (see appendix V), without any major protocol violations which would significantly compromise the integrity of collected data.

The Modified Exploratory Population: All participants from the exploratory population (see appendix V) with a negative C&S result.

11.3. Analysis Plan

11.3.1. Analysis Population

The Mod-ITT Population will be used for the analysis of the primary and secondary endpoints (exception for the discordance study, where the ITT population will be used for assessing the agreement between methods).

For the exploratory endpoint analysis, the Modified Exploratory Population will be used.

11.3.2. Primary, Secondary and Exploratory Endpoints

The primary endpoint is the number (and percentage) of patients in each study arm with FCl at the EOS visit; this term is defined as a *clinical response of improvement* and/or *clinical cure*:

Clinical response of improvement is defined as:
1) Resolution of cUTI signs and symptoms present at baseline, and/or
2) Development of no new cUTI symptoms, and/or
3) Avoidance of parenteral antibiotic therapy, in or out of hospital, at any time after randomization

Clinical cure is the resolution of all acute signs and symptoms of cUTI and improvement to such an extent that no further antimicrobial therapy (IV or oral) is required for the treatment of the cUTI

The secondary endpoints include:

- Number (and percentage) of patients in each arm with *microbiological eradication* of all baseline pathogens at the EOS visit; *microbiological eradication* of all baseline pathogens is defined as an end of study quantitative urine culture shows all uropathogens found at baseline are reduced to $<10^5$ CFU/mL and all baseline pathogens are not detected by EOS urine PCR (Cq>33)
- Subjective measurement of Treating Investigator Satisfaction Score as evaluated by the questionnaire at EOS
- Turnaround Time (TAT: as measured in hours) of molecular diagnostic procedures compared to conventional diagnostics (time from collection of samples to complete identification of organism(s) and availability of antibiotic sensitivity results)
- Overall agreeability between the diagnostic results generated by PCR versus C&S as assessed by discordant analysis Assess the favorable clinical outcomes of patients with discordant results [PCR(+), CS(-) and PCR(-), CS(+)]

- **<u>Exploratory Endpoints:</u>**

The negative percent agreement of the PCR test (specificity) compared to C&S in not detecting clinically insignificant UTIs

11.3.3. Statistical Analysis of the Primary Endpoint

The primary endpoint (percentage of patients exhibiting *favorable clinical outcomes*) will be evaluated for both arms of the study. A one-tailed, independent proportions Fisher's exact-test will be used to compare the PCR test procedure against the conventional (C&S) diagnostic procedure; the following hypotheses serve as the basis for the test:

$$H_0: p_{PCR}^{FCl} \leq p_{C\&S}^{FCl}$$
$$H_1: p_{PCR}^{FCl} > p_{C\&S}^{FCl}$$

where FCl indicates favorable clinical outcome (primary endpoint) as defined in the earlier sections of this protocol, PCR represents the test procedure and C&S represents the conventional procedure. A 95% confidence interval will be calculated with the test statistic. A p-value of less than 0.05 will trigger the rejection of the null hypothesis.

Further Data analysis investigation will be conducted by stratifying the data by various factors, that include but are not limited to the following:

- Study Sites (minimum 6 with n = 120)
- Age: Elderly (≥ 65yrs) versus Non-Elderly (< 65yrs)
- Gender: Male versus Female
- Comorbidities: High Risk (reporting comorbidities) versus Low Risk (no reported comorbidities)
- Pregnancy: Pregnant (positive for test) versus Non-Pregnant (negative for test or not applicable)

11.3.4. Statistical Analysis of Secondary Endpoints

Descriptive and trend statistics will be performed for the secondary variables. Continuous variables will be presented with the average and standard deviation or median range, as appropriate, and categorical variables will be presented as proportions. An EDA will be performed for the secondary and exploratory variables (except safety) to analyze and investigate data sets and summarize their main characteristics, employing data visualization methods where applicable. Exploratory subgroup analysis will be based on the potential prognostic variables, including but not limited to the strata described in section 11.3.4. All statistical significance tests will be conducted with an alpha of 0.05, unless specified otherwise. If the underlying population is observed to deviate significantly from normality, a non-parametric statistical approach will be used.

The level of discordance between the molecular method and the conventional method will be assessed by calculating the global study positive percent agreement (PPA) and negative percent agreement (NPA) from the results of the cUTI tests. It is noted that evaluating and incorporating the conventional method's inherent performance characteristics is out of scope

for this study; hence, the NPA/PPA metrics serve only as estimates into the sensitivity and specificity of the molecular method.

11.3.5. Statistical Analysis for the Exploratory Endpoints:

The negative percent agreement of the PCR test (specificity) in not detecting clinically insignificant UTIs will be calculated based on the C&S results.

12. PROTOCOL DEVIATION(S)

Protocol Deviation (PD) forms must be filed for an unintended or planned (if any and if approved in prior to occur) deviation from this protocol. PDs which place patients at increased risk of harm, or affect data integrity may be considered Protocol Violations (PVs) and must be reported to the appropriate regulatory authorities no later than 2 weeks (10 business days) from the time of identification. PDs will be filed in the applicable patient chart, and in the Trial Master File (TMF) upon study closeout. PDs will be filed and signed by delegated study staff and reviewed and signed by the Investigator. The Investigator will determine if the PD is reportable to the IRB based on an assessment of the patient's safety and/or the effects on the integrity of the study data.

13. PROTOCOL CHANGES

Any changes to the protocol must be tracked and documented in accordance with Good Documentation Practices, applicable SOPs, and regulatory oversight. The reasons for change must be documented in writing and provided to the regulatory bodies and approval must be granted prior to implementation unless subject safety is at risk. All versions of the protocol must be included in the TMF. All protocol changes will be documented in the study report.

14. ETHICAL CONSIDERATIONS

14.1. Ethical Conduct of the Study

The study will be performed in accordance with ethical principles that are consistent with the ICH guidelines for Good Clinical Practice (ICF GCP E6(R2)), applicable regulatory requirements and the Sponsor's policy on Bioethics.

14.2. IRB Approval

All necessary forms, advertisements, and subject-facing study documents will be compiled into a submission to an Institutional Review Board (IRB) for approval prior to the conduct of the study.

No conduct of the study will commence until written approval has been obtained from the IRB. The Sponsor and/or CRO must adhere to the requirements of the IRB and notify them of any study document changes, protocol amendments, and reportable protocol deviations/violations. Study termination must be reported to the IRB, and renewal of study approval must be obtained annually (or as per IRB's stipulations).

14.3. Informed Consent Form (ICF)

Informed consent will be obtained from patients by delegated study staff. The staff will explain the study and review each page of the consent document. Patients will then be given ample time to review the Informed Consent Form and ask any questions before signing the appropriate section(s). The Informed Consent Form (ICF) will contain pertinent study details, a statement indicating the patient is free to withdraw from the study at any point and for any reason, contact information of the IRB (to report ethical concerns), local and applicable regulations surrounding disclosure of personal and health information of the patients, and a section explaining the potential risk(s) of participating in the study. A copy of the informed consent document will be given to the patient for their records. The informed consent process will be documented in the source document (including the date), and the form signed, before the patient undergoes any study-specific procedures.

14.4. Risks and Procedures to Minimize Risk

Potential risks are disclosed to the study patients in the ICF prior to their participation in the study. The risk of the therapeutic intervention is judged comparable in comparison to standard of medical care. The proposed investigations and management strategies are all part of routine optimal care. The identifiable risks are associated with cUTIs. These risks include but are not limited to:

- Allergic reaction to antimicrobial therapy
- Progression or relapse of cUTI
- Nausea, vomiting, headache, skin rash, hypersensitivity reaction, anaphylaxis reaction
- Yeast infection (vaginitis/vulvovaginal candidiasis)
- Progression to acute renal failure
- C. difficile infection
- Sepsis

There may be unknown risks.

While there is minimal potential risk of loss of privacy and loss of confidentiality, multiple precautions will be in place to protect the subject's privacy.

15. QUALITY ASSURANCE AND QUALITY CONTROL

15.1. Auditing and Inspecting

The Sponsor, or designee(s), will monitor the study to ensure that the rights and well-being of the subjects are protected, for compliance with the protocol, for compliance with applicable laws and regulations. Quality assurance audits may be performed by the Sponsor or health authority during the course of the study or after its completion.

The PI and sites agree to comply with the Sponsor and regulatory requirements for auditing the study. This includes access to the source documents for source data verification.

15.2. Study Monitoring

Prior to the start of the study, the Sponsor representatives, site personnel, and any third-party vendor representatives will hold at least one meeting to go over the details of the study design and plans for study execution. The delegated study monitor will conduct risk-based monitoring to identify, assess, and mitigate the risks that could affect the quality or safety of a study. Monitoring and data verification may be performed remotely.

15.2.1. Responsibilities of the Investigator(s)

The Investigator must ensure compliance with all procedures required by the study protocol and with all study procedures provided by the Sponsor. The Investigator agrees to provide accurate study data requested by the study protocol (with the help of the CRF or other appropriate documents). If any process includes transfer of data, all protective measures should be in place to protect and maintain patient confidentiality while data is being transferred. The Investigator may appoint other individuals who may be deemed appropriate as Sub-investigators to assist in the conduct of the clinical study. All study staff will be delegated in a timely manner and listed in the TMF. The Investigator will provide all study staff with a copy of the clinical study protocol, ICF, ancillary forms, and all necessary details prior to delegation.

15.2.2. Responsibilities of the Sponsor

The Sponsor, or responsible CRO, of this clinical study is responsible for ensuring the proper conduct of the clinical study with regards to ethics, clinical study protocol compliance, and integrity and validity of the data recorded on the CRFs.

15.2.3. Source Document Requirements

The monitoring team will check the CRF/eCRF data against the source documents to verify accurate transfer of data, except for those entries captured directly into the CRF/eCRF as stipulated in the Clinical Trial Monitoring Plan (CTMP).

16. DATA HANDLING AND RECORD KEEPING

All US-based study sites and laboratories providing support for this study, must, where applicable, comply with the Health Insurance Portability and Accountability Act of 1996 (HIPAA). A site that is not a Covered Entity as defined by HIPAA must provide documentation of this fact to the CRO/Sponsor.

An independent, third-party clinical trial management system (CTMS) and/or Electronic Data Capture (EDC) software vendor may be used at the site for laboratory report data and patient-associated document storage. All activities and actions performed on the vendor platform(s) will be tracked and will produce an electronic audit-trail in accordance with regulatory standards.

Paper documents will be used in the event that the CTMS and/or EDC system(s) is not used due to any unforeseen or incidental circumstance causing disruption to data collection. All paper documentation will be subject to ICH-GCP E6(R2) regulations and applicable guidelines.

The PI and/or delegated study staff agree to maintain accurate CRFs and source documentation. Source documents are the originals of any documents that allow verification of the existence of the patient and substantiate the integrity of the data collected during the study.

The CRO and/or Sponsor will supply the site with either paper or electronic CRFs for each patient. CRFs will be completed only by persons delegated by the PI. Corrections will be made so as not to obliterate original data and will be identified and dated by the person who made the correction. The PI/delegates will allow designated representatives and regulatory bodies to have direct access to the source documents to verify the data reported in the CRFs.

16.1. Confidentiality

The PI and/or delegated study staff will ensure that the confidentiality of the patient's data will be preserved to the extent permitted by law. All parties will ensure protection of patient personal data and will not include names or other identifiable patient data in any reports, except where required by law. Documents that house patient information, such as signed ICFs and personal information/demographic forms will be maintained and stored by delegated study staff under strict access.

16.2. Source Data and Source Documents

As defined by the International Conference on Harmonization (ICH), source data are defined as all information in original records and certified copies of original records of clinical findings, observations, or other activities in a clinical study necessary for the reconstruction and evaluation of the study.

All source data and source documents will be stored and archived according to local regulatory requirements. For this study, source data and documents include, but are not limited to:

- Signed and dated Informed Consent Form (ICF);
- Name, sex, date of birth and other personal information/demographic information;
- Subject ID;
- Date and time of each visit;
- All clinical measurements and laboratory results;
- Status of patient throughout the study;
- Any first-hand study-related data directly captured on paper and/or entered into eCRFs via tablet, computer or other electronic devices;
- List of concomitant medication;
- Adverse events or changes in health;
- Reason for discontinuation/withdrawal, if applicable.

16.3. Case Report Forms (CRFs)

Case report forms will be created following protocol finalization and approval to capture study data. These forms may be in the form of paper, or may be electronic (eCRFs). An eCRF system, provided by an independent third-party vendor, may be used to capture data. Prior to deployment of the study, the eCRF system will be validated and specified to address source documentation, in accordance with Sponsor and regulatory requirements.

16.4. Data storage and Access

Data will be entered into the source documents, checked for discrepancies and queried for any issues in accordance with site-approved SOPs. The sites are responsible for collecting and inputting data into the designated study database, either directly or transcribing from source documents. The database housing the eCRF input will be hosted by the eCRF vendor. All vendor data access and entry can only be performed by authorized users, using a unique user login and password. Login activity and data entry will be tracked in an automated audit trail. The Sponsor, site(s) and CRO will permit study-related monitoring, audits, IRB review, and regulatory inspections, providing direct access to source data/documents. Paper CRFs may also be

monitored for completion, queried through internal review by delegated study staff, identified items resolved and documented where applicable, and any required data from source documents will be entered into the final database prior to locking.

16.5. Data Quality Assurance

Data cleaning will be performed to check for completeness and consistency of data using system, programmed, and manual edit checks. Discrepancies in data will be resolved through querying, delegation and resolution in accordance to site SOPs.

17. REFERENCES

1. Szlachta-McGinn A, Douglass KM, Chung UYR, Jackson NJ, Nickel JC, Ackerman AL. Molecular Diagnostic Methods Versus Conventional Urine Culture for Diagnosis and Treatment of Urinary Tract Infection: A Systematic Review and Meta-analysis. Eur Urol Open Sci. 2022;44:113-124. Published 2022 Sep 2. doi:10.1016/j.euros.2022.08.009
2. Pirkani GS, Awan MA, Abbas F, Din M. Culture and PCR based detection of bacteria causing urinary tract infection in urine specimen. Pak J Med Sci. 2020;36(3):391-395. doi:10.12669/pjms.36.3.1577
3. Bader MS, Hawboldt J, Brooks A. Management of complicated urinary tract infections in the era of antimicrobial resistance. Postgrad Med. 2010;122(6):7-15. doi:10.3810/pgm.2010.11.2217
4. Kande S, Patro S, Panigrahi A, Khora PK, Pattnaik D. Prevalence of uropathogens and their antimicrobial resistance pattern among adult diabetic patients. Indian J Public Health. 2021;65(3):280-286. doi:10.4103/ijph.IJPH_1413_20
5. Paul R. State of the Globe: Rising Antimicrobial Resistance of Pathogens in Urinary Tract Infection. J Glob Infect Dis. 2018;10(3):117-118. doi:10.4103/jgid.jgid_104_17
6. Simmering JE, Tang F, Cavanaugh JE, Polgreen LA, Polgreen PM. The Increase in Hospitalizations for Urinary Tract Infections and the Associated Costs in the United States, 1998-2011. Open Forum Infect Dis. 2017;4(1):ofw281. Published 2017 Feb 24. doi:10.1093/ofid/ofw281
7. Anger J, Lee U, Ackerman AL, et al. Recurrent Uncomplicated Urinary Tract Infections in Women: AUA/CUA/SUFU Guideline. J Urol. 2019;202(2):282-289. doi:10.1097/JU.0000000000000296
8. Efficacy and Safety Study of Eravacycline Compared with Levofloxacin in Complicated Urinary Tract Infections. ClinicalTrials.gov. https://clinicaltrials.gov/ct2/show/results/NCT01978938?term=Complicated+Urinary+Tract+Infections+outcomes&draw=2&rank=5. Updated Jan 11, 2022. Accessed May 12, 2023.

18. APPENDICES

Appendix I. Treating Investigator Questionnaire

*To be completed in reference to both urine PCR and C&S results	
Which test result were you provided with to use for your patient care?	☐ Urine PCR Result<br>☐ Urine C&S Result
Which of following describes your patient's cUTI event?	☐ Monomicrobial infection<br>☐ Polymicrobial infection<br>☐ Recurrent infection<br>☐ No infection
What was the clinical outcome of your patient care at EOS visit?	☐ Clinical improvement<br>(A complete resolution or significant improvement of signs or symptoms of cUTI)<br>☐ Clinical failure<br>(Your patient has 1) on-going AEs/SAEs at EOS and/or 2) persistent cUTI symptoms and/or 3) developed new symptoms of cUTI)<br>☐ Indeterminate<br>(if the outcome was other than clinical improvement or clinical failure. Provide reason: ____)<br>☐ N/A<br>(if the subject did not complete the study visit)
What was the microbiological outcome of your patient care at EOS visit? (eradication of the pathogens identified at baseline)	☐ Eradication<br>☐ Persistence<br>☐ Emergent infection (new infection or super-infection)<br>☐ Other (specify: ______________________________)
Based on provided diagnostic test results (PCR or C&S)	Score
1. Availability of diagnostic results (i.e. acceptable turnaround time to reduce the use of empiric broad-spectrum antibiotics)	(5) – Excellent<br>(4) – Good<br>(3) – Acceptable<br>(2) – Needs improvement<br>(1) – Unacceptable
2. Comprehensibility of diagnostic result interpretation	(5) – Excellent<br>(4) – Good<br>(3) – Acceptable<br>(2) – Needs improvement<br>(1) – Unacceptable

3. Efficiency of diagnostic process (i.e. usefulness of the test results in clinical decision making)	(5) – Excellent<br>(4) – Good<br>(3) – Acceptable<br>(2) – Needs improvement<br>(1) – Unacceptable
4. Overall satisfaction with the assigned diagnostic test	(5) – Very satisfied<br>(4) – More than satisfied<br>(3) – Satisfied<br>(2) – Partly satisfied<br>(1) – Not at all satisfied
5. Impact on patient care: Compared to the comparator test, clinical decision made with the assigned test result has yielded better patient clinical outcome	(5) – Strongly agree<br>(4) – Agree<br>(3) – Neither agree nor disagree<br>(2) – Disagree<br>(1) – Strongly disagree
Total Score:	/25

<u>Score Interpretation:</u>

Weighted summary scores in each criterion range from 1 to 5, with higher scores indicating greater diagnostic and therapeutic value of the specified test. A higher total score indicates better clinical utility of the assigned test than the comparator test.

Total score will be interpreted as follows:
The test method with a total score:

≥ 20	=	Good clinical utility
10 – 19	=	Moderate clinical utility
0 – 9	=	Poor clinical utility

Appendix II. Urine Sample Collection

- Early morning urine specimens ('first void specimen') are preferred, however routine or random samples are acceptable.
- Upon sample collection, immediately refrigerate the specimen and submit to the central laboratory within 24 hours of collection (maintain at 2-8 ºC when transporting).
- If transportation to the central laboratory is expected to exceed 24 hours, whole cups of urine specimen must be frozen. However, for expedited PCR test processing, aliquot 1mL of urine into two* separate 1.5mL Non-Stick RNase-Free microfuge tubes labeled with patient information. Aliquoted tubes (for PCR testing) and the remaining urine specimen in the cup (for C&S testing) must be place in the freezer (< -20ºC).

*One tube is for immediate processing on the following work day and the other is a backup in case testing needs to be repeated.

Methods	Supplies	Instruction
Clean-catch Midstream	- Sterile 90 mL container<br>- Towelette	*<u>Patient instructions:</u>*<br>Step 1. Wash and dry your hands thoroughly.<br>Step 2. Remove the container lid and set it aside. Do not touch inner surfaces.<br>Step 3. Cleanse your urogenital area ("lower parts") with the towelette provided.<br>Women: Wipe from front to back between the folds of skin. Continue to hold the skin fold apart until the urine sample is collected<br>Men: Wipe the tip of the penis (if un-circumcised, retract the foreskin). Continue to hold the foreskin back until the urine sample is collected<br>Step 4. Pass a small amount of urine into the toilet bowl.<br>Step 5. Urinate into the container until the container is 1/2 to 2/3 full<br>Step 6. Finish urinating into the toilet bowl.<br>Step 7. Replace the lid and tighten firmly.<br>Step 8. Wash and dry your hands thoroughly.

Note: The collector must NOT combine urine collected from separate voids to create one specimen of sufficient volume, under any circumstances.

Appendix III. Device specification: pathogen targets and Cq ranges, antibiotics resistance genes and Cq ranges

<table>
<tr><td>Pathogen targets
Candida albicans, glabrata, parapsilosis, tropicalis
Chlamydia trachomatis
Citrobacter freundii/braakii
Citrobacter koseri
Enterococcus faecium, faecalis
Escherichia coli
Gardnerella vaginalis
Klebsiella pneumoniae/oxytoca
Mycoplasma genitalium
Neisseria gonorrhoeae
Proteus mirabilis, vulgaris
Pseudomonas aeruginosa
Serratia marcescens
Staphylococcus (coagulase negative: epidermidis, haemolyticus, lugdunensis, saprophyticus)
Staphylococcus aureus
Staphylococcus saprophyticus
Streptoccoccus agalactia (group B)
Streptococcus pyogenes
Trichomonas vaginalis
Ureaplasma urealyticum
Acinetobacter baumannii</td><td>Cq ranges for pathogen detection

Critically High: 10.000 – 21.000
High: 22.000 – 26.000
Medium: 27.000 – 28.000
Low: 29.000 – 32.000
Negative: 40.000 – 33.000;
09.000 – 1.000</td></tr>
<tr><td>Antibiotic resistance genes
Class A ß-lactamase; blaKPC
Class A ß-lactamase; CTX-M-Group1
Class B metallo-ß-lactamase; blaNDM
Class D oxacillinase OXA-48
Class D oxacillinase OXA--51
dfr (A1, A5), sul (1,2) probes (Sulfamethoxazole and trimethoprim)
ermB, C; mefA
IMP, NDM, VIM Groups (Carbapenem)
MRSA* Mec-A
PER-1/VEB-1/GES-1 Groups (ESBL)
qnrA1, A2
qnrB
qnrS
tetB, tetM
VanA, VanB (Vancomycin)
ACT, MIR, FOX, ACC Groups (Beta Lactams)</td><td>Cq ranges for resistance gene identification

Positive: 10.000 – 31.000
Negative: 32.000 – 40.000;
1.000 – 9.000</td></tr>
</table>

Appendix IV. Comparator test results

1. PCR Positive test result: listings of detected microorganisms and antimicrobial sensitivity (sample)

Result Summary

Organism(s) Tested - Detected:

Organism Detected	Est. Microbial Load*	Total % Pathogen Load	Potential Therapeutic Agents
Escherichia coli	16.384*10^5 copies/mL	99.2248062%	- Amikacin IV<br>- Ciprofloxacin po/IV<br>- Ertapenem IV<br>- Levofloxacin po/IV<br>- Meropenem IV<br>- Doripenem<br>- Moxifloxacin<br>- Ofloxacin<br>- Plazomicin<br>- Levofloxacin<br>- Tobramycin<br>- Gentamicin<br>- Ciprofloxacin<br>- Meropenem
Gardnerella vaginalis	1.28*10^4 copies/mL	0.7751938%	- Doripenem<br>- Linezolid<br>- Clindamycin<br>- Metronidazole<br>- Clindamycin PO<br>- Linezolid PO<br>- Metronidazole (IV/po)

Antibiotic Resistance Detected:

Resistance Gene Detected	Resistant Against
Class A ß-lactamase; CTX-M-Group1	
dfr (A1, A5), sul (1,2) probes (Sulfamethoxazole and trimethoprim)	- Bactrim
ermB, C; mefA	- azithromycin<br>- clarithromycin<br>- erythromycin<br>- spiramycin
tetB, tetM	

2. PCR Negative test result (sample)

Result Summary

Organism(s) Tested - Detected:

No organisms detected

Antibiotic Resistance Detected:

No resistance detected

Urinary Tract Infectious Disease Pathogens

Organism	Results	Est. Microbial Load*
Candida albicans, glabrata, parapsilosis, tropicalis	Not Detected	Negative
Chlamydia trachomatis	Not Detected	Negative
Citrobacter freundii/braakii	Not Detected	Negative
Citrobacter koseri	Not Detected	Negative
Enterococcus faecium, faecalis	Not Detected	Negative
Escherichia coli	Not Detected	Negative
Gardnerella vaginalis	Not Detected	Negative
Klebsiella pneumoniae/oxytoca	Not Detected	Negative
Mycoplasma genitalium	Not Detected	Negative
Neisseria gonorrhoeae	Not Detected	Negative
Proteus mirabilis, vulgaris	Not Detected	Negative
Pseudomonas aeruginosa	Not Detected	Negative
Serratia marcescens	Not Detected	Negative
Staphylococcus (coagulase negative: epidermidis, haemolyticus, lugdunensis, saprophyticus)	Not Detected	Negative
Staphylococcus aureus	Not Detected	Negative
Staphylococcus saprophyticus	Not Detected	Negative
Streptococcus agalactia (group B)	Not Detected	Negative
Streptococcus pyogenes	Not Detected	Negative
Trichomonas vaginalis	Not Detected	Negative
Ureaplasma urealyticum	Not Detected	Negative
Acinetobacter baumannii	Not Detected	Negative

Antibiotic Resistance

Resistance Gene(s)	Antibiotic Class	Results	Est. Microbial Load*
Class A ß-lactamase; blaKPC	N/A	Not Detected	N/A
Class A ß-lactamase; CTX-M-Group1	N/A	Not Detected	N/A
Class B metallo-ß-lactamase; blaNDM	N/A	Not Detected	N/A
Class D oxacillinase OXA-48	N/A	Not Detected	N/A
Class D oxacillinase OXA--51	N/A	Not Detected	N/A
dfr (A1, A5), sul (1,2) probes (Sulfamethoxazole and trimethoprim)	N/A	Not Detected	N/A
ermB, C; mefA	N/A	Not Detected	N/A
IMP, NDM, VIM Groups (Carbapenem)	N/A	Not Detected	N/A
MRSA* Mec-A gene	N/A	Not Detected	N/A
PER-1/VEB-1/GES-1 Groups (ESBL)	N/A	Not Detected	N/A
qnrA1, A2	N/A	Not Detected	N/A
qnrB	N/A	Not Detected	N/A
qnrS	N/A	Not Detected	N/A
tetB, tetM	N/A	Not Detected	N/A
VanA, VanB (Vancomycin)	N/A	Not Detected	N/A
ACT, MIR, FOX, ACC Groups (Beta Lactams)	N/A	Not Detected	N/A

3. Urine C&S Positive test result: listings of detected microorganisms* and antimicrobial sensitivity (sample)

*The presented sample test report demonstrates a mere example of detectable microorganisms.

MICROBIOLOGY

Test Name	Results				Site
**Urine Culture**	**Status:**	**FINAL**	**06/20/22**	**10:55**	**IL**

Colony Count: >100,000 CFU/ml   Organism: **Escherichia coli**

	E coli	
ANTIBIOTICS	MIC	INTRP
**Amikacin**	<=16	S
**Amox/K Clav'ate**	<=8/4	S
**Amp/Sulbactam**	<=8/4	S
**Ampicillin**	<=8	S
**Aztreonam**	<=4	S
**Cefazolin**	<=2	S
**Cefepime**	<=8	S
**Cefotaxime**	<=2	S
**Ceftazidime**	<=1	S
**Ceftriaxone**	<=1	S
**Cefuroxime**	<=4	S
**Ciprofloxacin**	<=1	S
**Ertapenem**	<=0.5	S
**Gentamicin**	<=2	S
**Imipenem**	<=1	S
**Levofloxacin**	<=2	S
**Nitrofurantoin**	<=32	S
**Piperacillin/Tazo**	<=16	S
**Tetracycline**	<=4	S
**Ticar/K Clav'ate**	<=16	S
**Tobramycin**	<=4	S
**Trimeth/Sulfa**	<=2/38	S
**Trimethoprim**	<=8	S

S=SUSCEPTIBLE   I=INTERMEDIATE   R=RESISTANT

4: Urine C&S Negative test result (sample)

MICROBIOLOGY

Test Name	Results				Site
**Urine Culture**	**Status:**	**FINAL**	**06/19/22**	**10:14**	**IL**

06/19/22   ***No Growth***

Appendix V. Sub-study: Negative Patient Testing

<u>Sub-study Rationale:</u> The objective of this sub-study is to conduct a further assessment of the concordance between C&S tests and PCR probes. As an integral element of the clinical utility study, the clinical validation study plays a pivotal role in augmenting and strengthening the dataset. The planned sub-study is to verify that the PCR does not exhibit excessive sensitivity, thereby minimizing the likelihood of false positives, particularly those stemming from non-clinically significant UTI, when compared to the established standard of care in healthy patient population.

<u>Design/Plan:</u> Healthy individuals meeting the specified eligibility criteria and opting to take part in the sub-study will undergo the informed consent process. Following this, they will provide urine samples for both PCR and C&S testing. This sub-study is multi-center, non-randomized, single-arm, non-interventional study. The testing site settings will remain the same as in the main protocol (see *section 7.2.*), all adults patients attending medical visits to participating sites with no signs and symptoms of UTI will be offered to participate in the sub-study.

<u>Objective and Endpoint:</u>
The objective and endpoint for the sub-study are provided below.

Objective	Endpoint
• To assess the effectiveness of PCR in avoiding detection of clinically insignificant infections in comparison to the performance of C&S	• The negative percent agreement of the PCR test (specificity) compared to C&S in not detecting clinically insignificant UTIs

The safety objective of the main study is partially applicable as there are differences in visit description (see *section 4.).* Safety assessment for the sub-study population will be based on clinical observations, medical review of adverse event reports and relevant clinical laboratory tests at applicable study timepoints.

Study Population

The study population will consist of up to 360 individuals. No sample size calculation is required to conduct this sub-study.

<u>Inclusion Criteria:</u>
Subjects must meet all of the following criteria to be considered eligible for admission to the sub-study:

I1. At least 18 years of age at the time of consent;
I2. Not presenting any clinical signs and symptoms of a UTI (e.g., dysuria, frequency, urgency to urinate, burning sensations during urination, hematuria, suprapubic pain);

I3. Presenting a negative result on the urine dipstick test for both nitrite and leukocyte esterase
I4. Able to provide at least 8 mL urine

<u>Exclusion Criteria:</u>
Subjects meeting any of the following criteria will not be eligible for admission to the sub-study:
E1. Unable or unwilling to provide written informed consent;
E2. Three or more episodes of acute uncomplicated UTI in the past 12 months
E3. Presenting with fever (temperature >38 degrees Celsius or >100.4 degrees Fahrenheit), flank pain, chills, or any other manifestations suggestive of urinary tract infection;
E4. Currently participating in, or has participated in an interventional clinical trial with an investigational product or device within 30 days prior to the Screening Visit;
E5. Currently on or chronic use of any antibiotics for any clinical indication within 48 hours prior to the visit;
E6. Pregnant women with known fetal congenital anomaly (e.g., genetic abnormality or major congenital malformation) based on antenatal ultrasound;
E7. Medical condition or other factor that in the judgment of the investigator might affect ability to comply with procedures.

<u>Study Visit:</u>
This sub-study is designed as a single-visit study for sample collection.

1) Obtaining informed consent – the delegated study staff will explain the study objectives and the process of participation in the research. The study staff will answer any questions the patient may have. Patients will then be given ample time to review the ICF and ask any questions before signing the appropriate section(s). The ICF will then be signed by the Investigator or delegate. A copy of the ICF will be provided to the patient for their records.
2) Eligibility assessment – the study staff will collect demographic information, relevant medical history, concomitant therapy, vital signs, and examination to verify the eligibility criteria.
    a. Sample collection – A urine sample will be collected as a part of eligibility assessment. The subject should be instructed to provide at least 8mL of urine using an acceptable method as per *section 9.1.*). The collected sample will be tested with urine dipstick first, and only the subject with negative result will be enrolled.
3) Sample labelling and preparation – Collected urine samples of enrolled subjects will be labeled and prepared for processing according to *section 9.1.*
4) Subject exit documentation – Upon providing the adequate urine sample, the subject is considered to have completed the test procedure and will exit the study.

5) Sample processing – The collected urine sample will be aliquoted and analyzed by both urine PCR and C&S in accordance with SOPs.
6) Laboratory result documentation – Once the test results become available, study staff must document all study findings using the designated eCRF and associated redacted lab reports.

Schema:

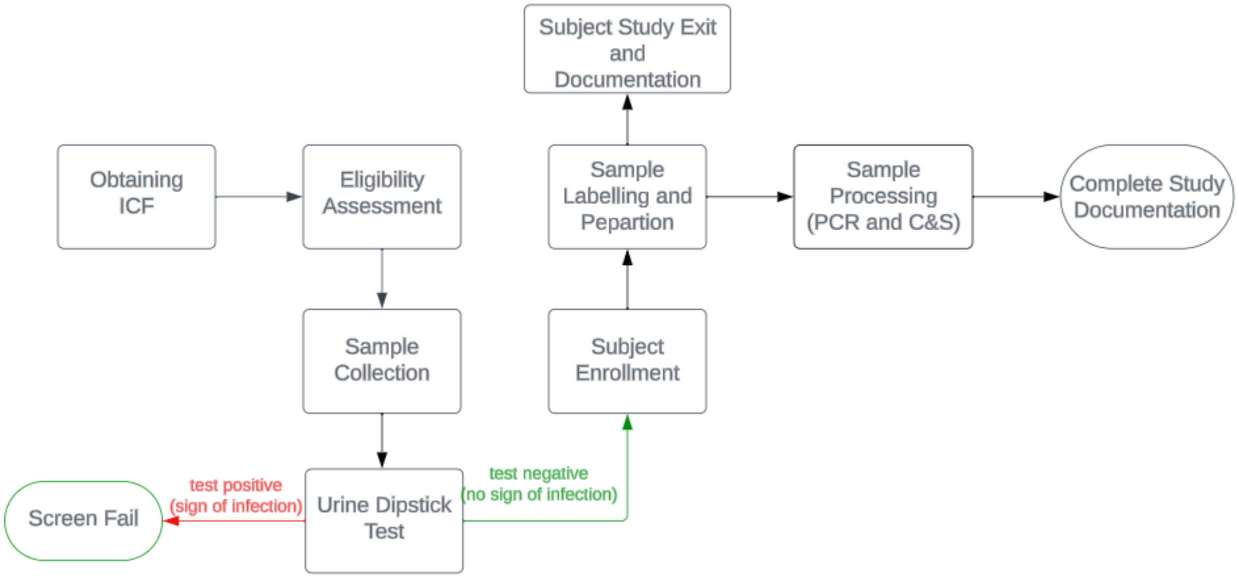

Subject Numbering System: For the sub-study population, subject IDs will adhere to the standardized format used for the main study population, but with the sequential enrollment number starting at 200.

Statistical Analysis: This sub-study endpoint will be analyzed as an exploratory endpoint within the main protocol. See *section 11*.
